# Computational Exploration of Potential Pharmacological Inhibitors Targeting the Envelope Protein of the Kyasanur Forest Disease Virus

**DOI:** 10.3390/ph17070884

**Published:** 2024-07-03

**Authors:** Sharanappa Achappa, Nayef Abdulaziz Aldabaan, Shivalingsarj V. Desai, Uday M. Muddapur, Ibrahim Ahmed Shaikh, Mater H. Mahnashi, Abdullateef A. Alshehri, Basheerahmed Abdulaziz Mannasaheb, Aejaz Abdullatif Khan

**Affiliations:** 1 Department of Biotechnology, KLE Technological University, Hubballi 580031, Karnataka, India; sharanappaa@kletech.ac.in (S.A.); muddapur@kletech.ac.in (U.M.M.); 2 Department of Pharmacology, College of Pharmacy, Najran University, Najran 66462, Saudi Arabia; 3 Department of Pharmaceutical Chemistry, College of Pharmacy, Najran University, Najran 66462, Saudi Arabia; matermaha@gmail.com; 4 Department of Clinical Laboratory Sciences, College of Applied Medical Sciences, Najran University, P.O. Box 1988, Najran 66462, Saudi Arabia; aaalshehre@nu.edu.sa; 5 Department of Pharmacy Practice, College of Pharmacy, AlMaarefa University, P.O. Box 71666, Riyadh 11597, Saudi Arabia; 6 Department of General Science, Ibn Sina National College for Medical Studies, Jeddah 21418, Saudi Arabia

**Keywords:** Kyasanur forest disease virus, envelope protein, molecular docking, molecular dynamic simulation, pharmacophore screening

## Abstract

The limitations of the current vaccination strategy for the Kyasanur Forest Disease virus (KFDV) underscore the critical need for effective antiviral treatments, highlighting the crucial importance of exploring novel therapeutic approaches through in silico drug design. Kyasanur Forest Disease, caused by KFDV, is a tick-borne disease with a mortality of 3–5% and an annual incidence of 400 to 500 cases. In the early stage of infection, the envelope protein plays a crucial role by facilitating host–virus interactions. The objective of this research is to develop effective antivirals targeting the envelope protein to disrupt the virus–host interaction. In line with this, the 3D structure of the envelope protein was modeled and refined through molecular modeling techniques, and subsequently, ligands were designed via de novo design and pharmacophore screening, yielding 12 potential hits followed by ADMET analysis. The top five candidates underwent geometry optimization and molecular docking. Notably, compounds L4 (SA28) and L3 (CNP0247967) are predicted to have significant binding affinities of −8.91 and −7.58 kcal/mol, respectively, toward the envelope protein, based on computational models. Both compounds demonstrated stability during 200 ns molecular dynamics simulations, and the MM-GBSA binding free-energy values were −85.26 ± 4.63 kcal/mol and −66.60 ± 2.92 kcal/mol for the envelope protein L3 and L4 complexes, respectively. Based on the computational prediction, it is suggested that both compounds have potential as drug candidates for controlling host–virus interactions by targeting the envelope protein. Further validation through in-vitro assays would complement the findings of the present in silico investigations.

## 1. Introduction

The Kyasanur Forest Disease virus (KFDV), responsible for causing Kyasanur Forest Disease (KFD), which induces a hemorrhagic illness in humans, belongs to the Flaviviridae family and was first recognized in 1957 as a febrile illness in Shivamogga, Karnataka, India [1,2]. KFDV primarily targets the black-faced langur (*Presbytis entellus*) and the red-faced bonnet monkey (*Macaca radiata*). It has a zoonotic origin and spreads through the bites of infected ticks, especially during their nymphal stage, with these ticks remaining infectious throughout their lifespan. KFDV, often colloquially referred to as “monkey fever”, circulates among smaller animals, including rodents, shrews, and birds [3]. While adult ticks prefer larger animals as their hosts for reproduction, they do not exhibit symptoms of the disease. These ticks act as the primary hosts and reservoirs for KFDV, while monkeys serve as secondary hosts, and humans act as dead-end hosts for the virus. KFDV is a zoonotic and vector-borne virus circulating between vector ticks and vertebrate hosts capable of acting as reservoirs. Various vertebrate species, such as monkeys, shrews, bats, birds, and small rodents, participate in its transmission cycle [4]. Annually, approximately 400–500 new human cases of KFD are reported, with a mortality rate ranging from 3% to 5% [3,5]. The United States National Institute of Allergy and Infectious Diseases (NIAID) has designated this virus as a category C priority pathogen, primarily based on its significant pathogenicity and the lack of licensed vaccines and therapies by the US Food and Drug Administration (FDA). Furthermore, the infectious dose for KFDV remains unknown [4,6]. Following internationally recognized biosafety regulations, KFDV is categorized as a risk group 4 pathogen due to its unpredictable ecological and epidemiological features. In the International Classification of Diseases-10 (ICD-10) from 2017, KFD is classified under category A98.2, which falls within the broader category of other viral hemorrhagic fevers not otherwise classified [7].

Epidemiological investigations have consistently been conducted to monitor and address disease outbreaks in the Shivamogga district of Karnataka, India, usually occurring seasonally from November to June [8]. Over the past 15 years, there has been a noticeable rise in outbreaks extending beyond the customary endemic areas. It is worth mentioning that during the period from 2012 to 2014, the first significant occurrences of KFD were recorded in regions that are not traditionally affected by the disease. These areas include the Chamarajanagar district of Karnataka as well as the surrounding Malappuram and Wayanad districts of Kerala [4,5,9]. Likewise, during the years 2015 and 2016, occurrences of the abovementioned sickness were observed in the North Goa and Sindhudurg regions of Goa and Maharashtra, respectively [10,11,12]. In a recent development, an instance of KFD was detected in China, signifying the initial manifestation of KFDV beyond the borders of India [13]. Recognized as a neglected human pathogenic virus, the expanding endemic regions of KFDV raise concerns, highlighting the urgent need for the development of new preventive measures and treatments. This underscores the importance of gaining a more profound understanding of the pathogenesis, ecology, and epidemiology of KFDV [14]. KFDV is characterized as a spherical virus enveloped in a lipid bilayer with an approximate diameter of 45 nm. Its genetic material comprises a single-stranded, positive-sense RNA genome spanning a total length of 10,774 nucleotides, and shares the same 45 nm diameter [15]. The polyprotein, encompassing 3146 amino acids, is divided into three structural proteins: capsid (C), precursor membrane (prM), and envelope (E), along with seven non-structural proteins—NS1, NS2A, NS2B, NS3, NS4A, NS4B, and NS5 [16].

In the initial stages of flavivirus invasion, the envelope protein assumes a pivotal role in facilitating connections between the virus and the host. It actively engages in membrane fusion and contributes to the unveiling of the virion [17,18]. Specifically, the envelope protein of flaviviruses, such as the dengue virus, adheres to the cell surface through factors like glycosaminoglycans, initiating virus entry through receptor-mediated, clathrin-dependent endocytosis [19]. The envelope protein, with its receptor-binding site and fusion peptide, helps the new viral genome assemble and release the virus from the host cell. [20]. Upon penetration into the endosome, the viral envelope protein undergoes structural changes prompted by the acidic environment, leading to the fusion of host endosomal membranes with viral membranes [21]. Throughout the life cycle of flaviviruses, virions undergo considerable structural modifications, ultimately maturing. During this transition, the dimeric form of the envelope protein interacts with human receptors, bolstering virion stability in a post-fusion state [22]. The dimeric envelope protein subsequently disassembles into monomers, later reassembling into trimeric forms [20,21]. The central domain I (DI) links the extended domain II (DII) with the globular domain III (DIII), playing an essential role in receptor binding. This structural aspect is consistent with other flaviviruses like dengue and Japanese encephalitis, where B-cell epitopes are predominantly situated in domains DI and DII [23].

The envelope protein also contains two glycosylated asparagine residues, Asn67 and Asn153, which are involved in interacting with cell surface receptors [24]. X-ray crystallography studies of the envelope protein of the dengue virus, when complexed with the DC Sign receptor, have shed light on the molecular mechanisms underpinning envelope protein interactions with host receptors during the entry of flaviviruses into host cells [25]. Notably, the envelope protein is responsible for triggering host immune responses, including the production of protective and neutralizing antibodies. This underscores its significance in drug development aimed at inhibiting the functionality of the E-protein [26]. The initial approach to KFDV prevention involved the use of a formalin-inactivated whole virus vaccine. However, this vaccine demonstrated limited efficacy [15,27]. Consequently, the future of this vaccine remains uncertain, leaving the public health response susceptible. The increasing number of KFDV cases and the expansion of endemic areas in India have raised significant concerns within the region’s public health infrastructure [28].

As the current vaccination strategy has proven to be less effective, and given the unavailability of effective antiviral treatments, the exploration of novel therapeutic approaches for KFDV is significant. This necessitates an interdisciplinary approach to comprehensively understand the virus, including its vectors, pathogenesis, and epidemiology. The objective of the research is to develop novel drugs by determining the 3D structure of the KFDV envelope protein using bioinformatics tools. Without existing 3D structures, we used Robetta and I-TASSER to predict the protein’s 3D structure and validate these models. We then employed both ligand-based and structure-based methods to identify potential drug candidates. Finally, the interactions between the developed drugs and the envelope protein were examined through molecular docking and molecular dynamics (MD) simulations, providing detailed insights into their efficacy and stability. By achieving these objectives, the research aims to pave the way for effective antiviral treatments for KFDV using the in silico approach.

## 2. Results

### 2.1. Sequence Analysis

**Figure 1:**
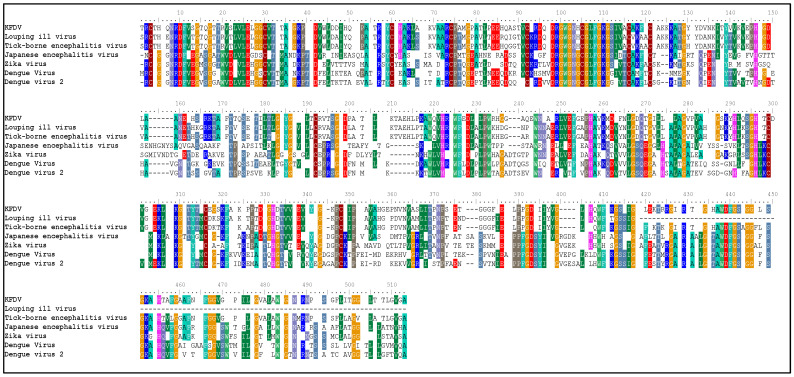
MSA between KFDV and homologous sequence.

The envelope protein sequence (282–777 aa) with UniProt ID D7RF80.1 was obtained from the biological database of UniProt. To identify homologous sequences, a BLASTp analysis was performed with the PDB as the database, using default algorithm parameters. Sequences with query coverage close to 100% and low e-values were considered. The BLASTp results are represented in Table 1. A multiple sequence alignment (MSA) was conducted between the target sequence D7RF80.1 and the structures of tick-borne encephalitis virus, dengue virus 2, Zika virus, Louping ill virus, and Japanese encephalitis virus (PDB ID: 5O6A, 4CBF, 5GZR, 6J5C, and 5WSN, respectively) using Clustal omega. The MSA revealed a high degree of conservation in most regions. Specifically, 12 cysteine residues at positions 3, 30, 60, 74, 92, 105, 116, 121, 186, 290, 307, and 338 were identified as conserved among all other flaviviruses. Furthermore, the formation of disulfide bonds between these cysteine residues, as shown in Figure 1, suggests a similar three-dimensional structure [29].

The physicochemical properties of the envelope protein were assessed using the ProtParam tool, and the findings have been organized in Table 2.

The isoelectric point (pI) of the protein represents the pH at which the protein becomes electrically neutral, stable, and compact and exhibits minimal solubility and mobility in an electro-focusing system [30]. In the case of the envelope protein, the computed theoretical pI was found to be 7.2, indicating its basic nature. The aliphatic index (AI) of the protein, which was measured at 83.10, suggests that the envelope protein is stable across a wide range of temperatures. This stability is attributed to the presence of a higher number of aliphatic side-chain amino acids, such as alanine, valine, isoleucine, and leucine [31]. The instability index (II) of the envelope protein was determined to be 29.41, which is below the threshold of 40. This value indicates that the protein is stable in a solution containing specific dipeptides [32]. Lastly, the GRAVY (grand average of hydropathicity) value was calculated and found to be −0.16. A negative GRAVY value implies that the protein is hydrophilic and has a favorable interaction with water molecules. This is especially relevant because the envelope protein is extracellular and interacts with the host immune system [33]. The secondary structure of the envelope protein was predicted using SOPMA (structure and function of biomolecules: parallelized in silico molecular analysis), and the results have been summarized in Table 3.

The secondary structure analysis of the envelope protein revealed that it is primarily composed of a random coil, accounting for 40.9% of the amino acid composition. Extended strands make up 33.67% of the secondary structure, while alpha helices constitute 19.15%. This distribution suggests that the protein can be classified as a mixed-class protein, as it contains a lower number of helices and strands in comparison with other secondary structural elements. Additionally, the transmembrane region of the envelope protein was predicted using TMHMM 2.0, which segmented the protein into distinct areas. The amino acids from 1 to 446 were identified as the extracellular region, amino acids 447 to 469 were classified as the transmembrane region, and amino acids 470 to 475 were designated as the cytoplasmic region. These findings are summarized in Table 4.

### 2.2. The 3D Structure Determination and Structure Validation

The 3D structure of the envelope protein was predicted using both the Robetta and I-Tasser servers. Robetta generated a model using the PD algorithm with a confidence score of 0.93. On the other hand, I-Tasser produced five models using templates from LOMETS from the PDB. The model with the highest c-score of 1.51, a TM-score of 0.92 ± 0.06, and an RMSD of 4.1 ± 2.8 Å was chosen for further analysis. To ensure the reliability of the selected models from Robetta and I-Tasser, various structure quality assessment software tools were employed. The results of this assessment are presented in Table 5.

**Figure 2:**
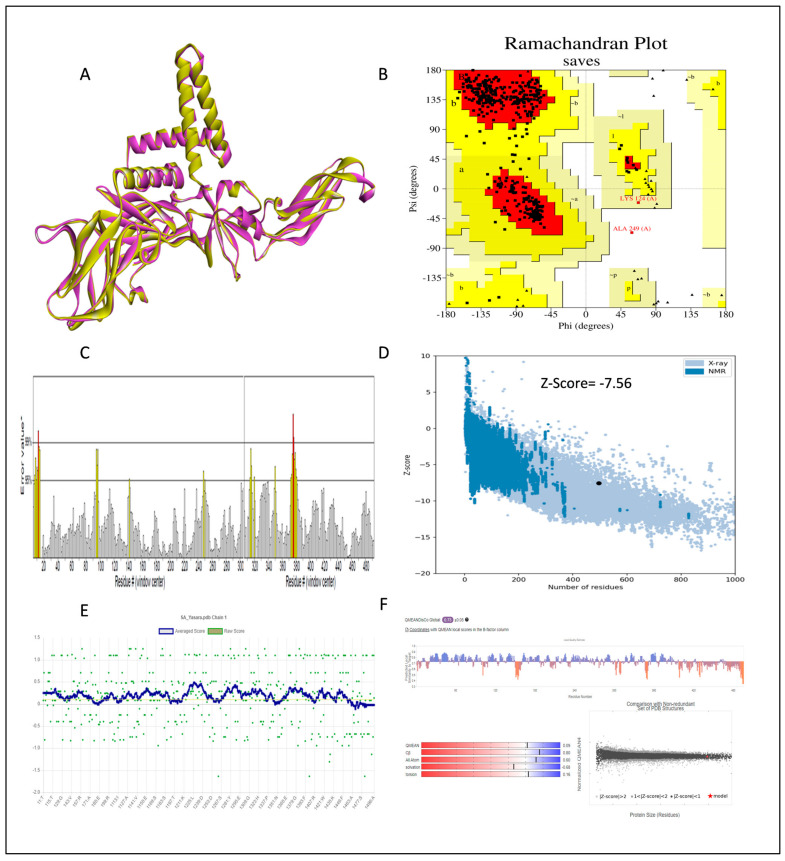
(**A**) Superimposed Robetta model (yellow color) and refined model (violet color) and validation of refined Robetta modeled envelope protein; (**B**) Ramachandran plot; (**C**) ERRAT quality factor; (**D**) ProSA Web score; (**E**) Verify3D score; and (**F**) Qmean score.

The stereochemical analysis of the model structure was conducted through a Ramachandran plot using the PROCHECK program, which classifies amino acid residues based on their psi and phi angles. The results of the Ramachandran plot revealed different regions within the model proteins, including the most favored region, additionally allowed region, generously allowed region, and disallowed region. The model predicted by I-Tasser had approximately 75.4% of its residues in the most favored region, indicating a somewhat lower stereochemical quality. In contrast, the model generated by Robetta exhibited a higher percentage, with 93.1% of amino acids falling within the favored region. This suggests that the Robetta model has better stereochemical quality and is more energetically favored. Validation analyses were performed using various tools. The Verify3D analysis showed that 89.11% of the residues in the Robetta model had a 3D-1D score of ≥0.2 on average, while the I-Tasser model scored 78.63%, failing to pass this validation. The ERRAT scores for both Robetta and I-Tasser were high, with 89.85 and 91.51%, respectively, indicating the high quality of their tertiary structures. The ProSA analysis further confirmed the quality of the models, with Z-scores falling within the range of those of PDB experimentally determined native structures. The amino acids’ local energy profile plot displayed mostly negative values across the sequence, except for the residue at the C-terminal, indicating a favorable amino acid energy profile and high accuracy and reliability of the models. Based on these validations, the Robetta model was chosen for further refinement using the YASARA energy minimization server. The energy calculated before energy minimization was −58,796.02 kcal/mol, and after energy minimization, it improved to −60,939.90 kcal/mol. The refined model also showed an improvement in the overall quality of the structure, with an increase in ERRAT score from 89.85 to 94.19. Figure 2 displays the validation of the refined Robetta model with a 3D structure.

### 2.3. Active Site Determination

An active site or binding site within a protein is a specific location where ligands can bind, and it plays a crucial role in drug design. The COACH-D server predicted the top two binding sites for the refined envelope protein, and these are detailed in Table 6. The best prediction had a high confidence score of 0.09, indicating very high reliability. Furthermore, the docking energy of the representative ligand and template complex was −4.6 kcal/mol, suggesting a strong preference and affinity for potential ligands at this site [34]. The second-ranked predicted binding site had a c-score of 0.07, which is higher than the lower-ranked binding sites. Table 6 also provides information about the binding residues for the top five predicted binding sites, along with their binding energy and grid box dimensions. COACH-D also identified a tentative ligand template for all the predicted binding sites. The ligand identified was Octyl-beta-D-glucopyranoside(BOG) (PubChem ID: 62852), which was subsequently used for ligand screening and pharmacophore pattern definition.

### 2.4. Ligand Design

#### 2.4.1. De Novo Drug Designing

De novo drug designing involves the creation of entirely new compounds from molecular fragments for the active site of the protein using computational algorithms, which is efficient, cost-effective, and offers improved biological activity [35,36]. The design criteria for these drugs include a molecular weight cutoff between 100 to 560, a log P value between 0 and 5, 1 to 10 hydrogen bond donors, 1 to 5 hydrogen bond acceptors, and the dimensions of the active site in XYZ coordinates obtained from COACH-D. To perform this, a genetic algorithm was employed, starting with a population of FDA-approved drugs and allowing for a maximum population size of 20 and 50 generations. A total of 50 drugs were designed using existing fragments in the database, and they were named from SA1 to SA50. The complete list of these 50 compounds designed by the eLea3d server can be found in Supplementary Table S1, and was subsequently used for virtual screening using PyRx 0.8 version software.

#### 2.4.2. Pharmacophoric Screening

**Figure 3:**
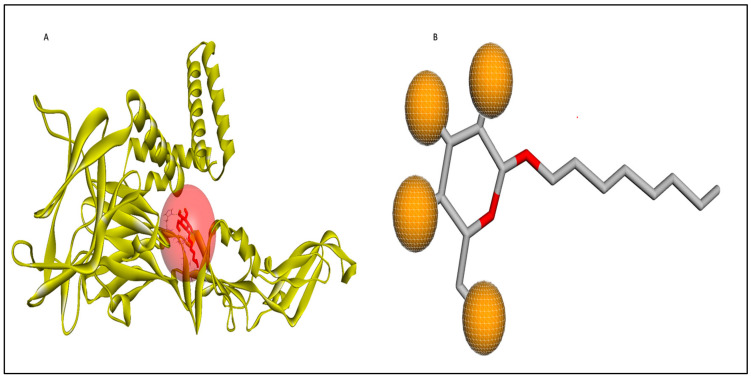
(**A**) Active site (Pink transparent sphere) of envelope protein with BOG ligand (Stick model, Red color) binding, (**B**) features of the generated pharmacophore model. The orange sphere indicate hydrogen bond acceptors, and the white dotted line pattern on orange sphere indicates a hydrogen bond donor site.

The Pharmit server was utilized to perform ligand-based and structure-based pharmacophore modeling to screen ligand databases based on the 3D structure of the ligand–protein complex [37]. In addition, structure-based pharmacophore models can also be generated with computationally derived ligand–target complexes. In the course of a docking run, known active compounds are fitted into the empty binding pocket of the target [38,39]. These docked binding poses can then be employed directly to extract the interaction patterns. For further refinement of the initial docking poses, molecular dynamics (MD) simulations can be conducted [40] prior to model generation. In the present study, the process began by extracting pharmacophore features from the predicted binding interactions and docked pose between the modeled envelope protein and BOG (PubChem ID: 62852), a known cofactor for dengue virus envelope protein [41]. This extraction was accomplished using Webina server 1.0.5, based on the AutoDock Vina algorithm [42]. The resulting pharmacophore model was then generated using the Pharmit server by uploading the modeled envelope protein as receptor and BOG as pharmacophore features. The pharmacophore features are detailed in Table 7 and visualized in Figure 3. To enhance the inclusivity of the search, “inclusive” shape constraints with a tolerance level of 1 were applied, ensuring that at least one heavy atom of the screened compounds aligned with the pharmacophore pose. Several other filters were also employed to identify compounds with a more “drug-like” profile. These filters included octanol-water partition coefficient log P values within the range of 0 to +5, a maximum of 10 hydrogen bond acceptors, a maximum of 5 hydrogen bond donors, and a molecular weight between 100 and 560. The selection of these parameter values adhered to the drug-likeness rules proposed by Lipinski [43]. The model was then used to search two extensive databases: the COCONUT library, which encompasses 8,739,148 conformers from 793,782 molecules, and the Zinc database, containing 122,276,899 conformers from 13,127,550 molecules. The search in the COCONUT database yielded 7374 hits, while the Zinc database produced 1869 hits. These hits were based on the pharmacophore pattern generated from the BOG drug molecule. Subsequently, the selected hits underwent binding energy calculations using the AutoDockVina scoring function within Pharmit.

#### 2.4.3. Ligand-Based Screening

PubChem, established in 2004 as part of the Molecular Libraries Roadmap Initiatives by the US National Institutes of Health (NIH), serves as a publicly accessible repository for information pertaining to chemical substances and their associated biological activities. PubChem encompasses three interconnected databases: Substance, Compound, and Bioassay. Utilizing the ligand obtained from COACH-D, we conducted a ligand-based screening against the PubChem database, applying a Tanimoto threshold of 90% and adhering to Lipinski’s Rule of Five. A total of 3808 drugs were subjected to this screening process and subsequently downloaded in SDF format for further utilization in virtual screening against the envelope protein, employing PyRx software.

### 2.5. Virtual Screening

The drug screening process involved both primary and secondary stages using PyRx with the AutoDock Vina set to an exhaustiveness of 8. Initially, a search in the PubChem database resulted in the identification of 3808 compounds for screening against the envelope protein. Subsequently, 33 compounds with binding energies ranging from −10.00 to −7.70 kcal/mol were chosen for secondary screening using PyRx. Further pharmacophore screening was conducted using the COCONUT and Zinc databases from Pharmit, yielding 7374 and 1869 compounds, respectively. Based on the minimum binding energy criteria, 73 compounds from the COCONUT database and 25 compounds from the Zinc database were selected for secondary screening. The list of selected compounds from the COCONUT and Zinc databases, based on their binding energy values, can be found in Supplementary Table S2. Additionally, a primary screening of 50 compounds, generated through de novo design using eLea3D, was carried out with PyRx, and 15 compounds exhibiting binding energy values in the range of −8.60 to −7.90 to kcal/mol were selected for secondary screening.

In the secondary screening phase, 146 drugs were assessed using PyRx against an energy-minimized envelope protein (33 compounds from PubChem, 73 compounds from the COCONUT database, 25 compounds from the Zinc database, and 15 compounds from the de novo design). The process involved ligand–receptor docking runs with Vina, which evaluated the results, calculated binding affinities, and organized the poses based on conformational overlaps. The best pose from each cluster was selected, and the ligands were ranked according to their binding affinities [44]. This screening identified 12 hits based on their binding affinities and orientations within the protein’s binding site, and these hits were further subjected to ADMET studies and molecular docking by AutoDock. A comprehensive summary of the binding affinities for the drugs, generated through various drug design and screening methods, can be found in Table 8, with additional information in Supplementary Table S3.

### 2.6. Toxicity Measurement

Prior to initiating clinical trials and in the pursuit of identifying superior lead compounds, it is imperative to conduct in silico toxicity assessments. The adoption of computer-based toxicity measurements has gained considerable popularity due to their precision, speed, and accessibility, enabling the evaluation of both synthetic and natural compounds. To evaluate the toxicity and possible adverse effects linked to the twelve chosen chemicals, we utilized the ProTox II server. The assessment encompassed different toxicological parameters, including acute toxicity, mutagenicity, cytotoxicity, hepatotoxicity, carcinogenicity, and the determination of the median lethal dose (LD50) in mg/kg, which was calculated based on weight and is presented in Table 9. As per the ProTox II server’s findings, compounds falling within class 4 or higher were identified and subjected to further evaluation for different types of toxicity. Among them, compounds 16178612, CNP0247967, SA8, ZINC0001000052673, CNP0097629.2, and CNP0247704.2, classified in class 4 and above, were predicted to have very low or no oral toxicity, carcinogenicity, mutagenicity, or cytotoxicity. On the other hand, compounds CNP0178494, SA28, and SA29, also belonging to class 4 or higher, were predicted to have mutagenic activity. The complete roster of compounds selected based on virtual screening data for toxicity prediction is provided in Table 9. Subsequently, relying on the ProTox II server results, compounds 16178612, CNP0247967, SA8, ZINC0001000052673, CNP0097629.2, and CNP0247704.2 were chosen for further analysis in the context of ADME (absorption, distribution, metabolism, and excretion) considerations.

### 2.7. ADME Analysis

An effective drug candidate must possess the capability to reach the target site within the body at concentrations that are optimized for therapeutic action [45]. The prediction of pharmacological and physicochemical parameters plays a crucial role as it provides insights into whether the molecule can attain the desired concentration at the active site and sustain it to trigger the intended biological response. The physicochemical and pharmacokinetic attributes of the six compounds were evaluated through SwissADME [45] and are presented in Table 10 for reference.

The physicochemical profiling included the assessment of both Lipinski’s Rule of Five (RO5) and Veber’s Rule to ascertain whether the chemical compounds with specific pharmacological or biological activities possess the necessary chemical and physical properties for oral activity [43,46]. With the exception of CNP0097629.2 (with a molecular weight exceeding 500), all compounds adhered to Lipinski’s Rule. As for Veber’s rules, compounds ZINC0001000052673, CNP0097629.2, and CNP0247704.2 violated one of the criteria, as summarized in Table 10. Lipophilicity, a property enabling drug-like compounds to dissolve in fats, oils, and nonpolar solvents, facilitating their diffusion through cell membranes, indicates the potential for oral administration. However, for compounds other than SA8 and CNP0247967, gastrointestinal absorption is low, suggesting that injectable dosage forms may be more effective in achieving a rapid onset of action. In terms of lipophilicity, all six compounds exhibited good properties, except for 161783612, which had a consensus log P value of −0.7, falling outside the range of +5.00. Solubility, a crucial physicochemical property, was predicted to evaluate the bioavailability and bioactivity of the identified compounds [47]. The poor solubility of lead compounds has led to their failure in clinical trials despite their potency, emphasizing the significance of early-stage solubility predictions in drug design [48]. Fortunately, all compounds were predicted to be soluble. The evaluation of molar refractivity (MR) provided valuable insights into the pharmacokinetics and pharmacodynamics of the compounds, considering various interactions in solution such as drug–solvent, drug–drug, and drug–co-solute interactions [49]. Except for SA8 and CNP0097629.2, all compounds fell within the acceptable range of MR values, ranging from 40 to 130. Topological polar surface area (TPSA) assessment indicated good oral bioavailability for all compounds, as their values did not exceed 140 Å^2^. However, ZINC0001000052673, CNP0097629.2, and CNP0247704.2 were exceptions, predicted to have lower oral bioavailability. Synthetic accessibility [50] was explored to evaluate the feasibility of synthesizing the de novo hits, with all compounds predicted to have easy synthesis. Many druggable candidates, especially de novo-constructed chemical entities, are unable to reach clinical trials due to their molecular complexity coupled with difficulty in synthesis [51].

SwissADME predicted that all six compounds, except 161783612 (L1), possess synthetic accessibility. An examination of potential non-specific reactivity with various biological targets identified PAINS (pan assay interference compounds) [52,53], and all compounds, except CNP0097629.2, were predicted not to contain PAINS substructures. Pharmacokinetics studies assessed parameters such as blood–brain barrier (BBB) permeability, gastrointestinal absorption (GI), and permeability glycoprotein (P-gp) interactions [54]. None of the compounds were predicted to cross the blood–brain barrier [55,56]. Ligands SA8 and CNP0247967 exhibited high GI absorption scores, while others displayed low GI absorption [57]. Additionally, all compounds, except ZINC0001000052673, were predicted to be P-glycoprotein substrates, potentially enhancing their bioavailability. In summary, the predictions suggest that all the compounds may exhibit favorable pharmacokinetic and pharmacodynamic profiles.

### 2.8. Molecular Docking

Molecular docking plays a crucial role in comprehending the interaction between ligands and proteins at the atomic level, facilitating the screening of drug molecules from libraries. Following ADME and toxicity assessments, AutoDock was employed to analyze the selected six compounds. The binding affinities of all six ligands fell within the range of −7.82 to −4.1 kcal/mol, as detailed in Table 11. Based on these binding affinities, five compounds/ligands were chosen for further geometric optimization, which was carried out using Orca version 4.2.1 software.

### 2.9. DFT Studies

Geometry optimization is a quantum chemical technique that involves refining rough geometric approximations to achieve the highest degree of precision [58]. The geometry characterized by the lowest energy state signifies the most stable form, as molecules tend to reduce their energy spontaneously. Consequently, utilizing Orca 4.2, we have determined the molecular geometry that is best optimized and possesses the lowest energy value. The optimized energy of the ligand structures determined by Orca is depicted in Table 12 for reference.

### 2.10. Frontier Molecular Orbital—HOMO/LUMO Calculation

The frontier molecular orbital (FMO) theory is vital in organic chemistry for understanding molecular structure and reactivity. It uses the HOMO-LUMO energy gap to reveal a molecule’s properties [59]. The gap between the highest occupied molecular orbital (HOMO) and the lowest unoccupied molecular orbital (LUMO) indicates sensitivity to reactions and stability. In the HOMO, electrons are engaged in nucleophilic reactions, while in the LUMO, electrons are involved in electrophilic reactions. Molecules with a narrow HOMO-LUMO gap are highly reactive and less stable (“soft”), while those with a wide gap are less reactive, more stable, and have lower bioactivity. A high FMO energy gap signifies better stability and lower reactivity [54,59].

**Figure 4:**
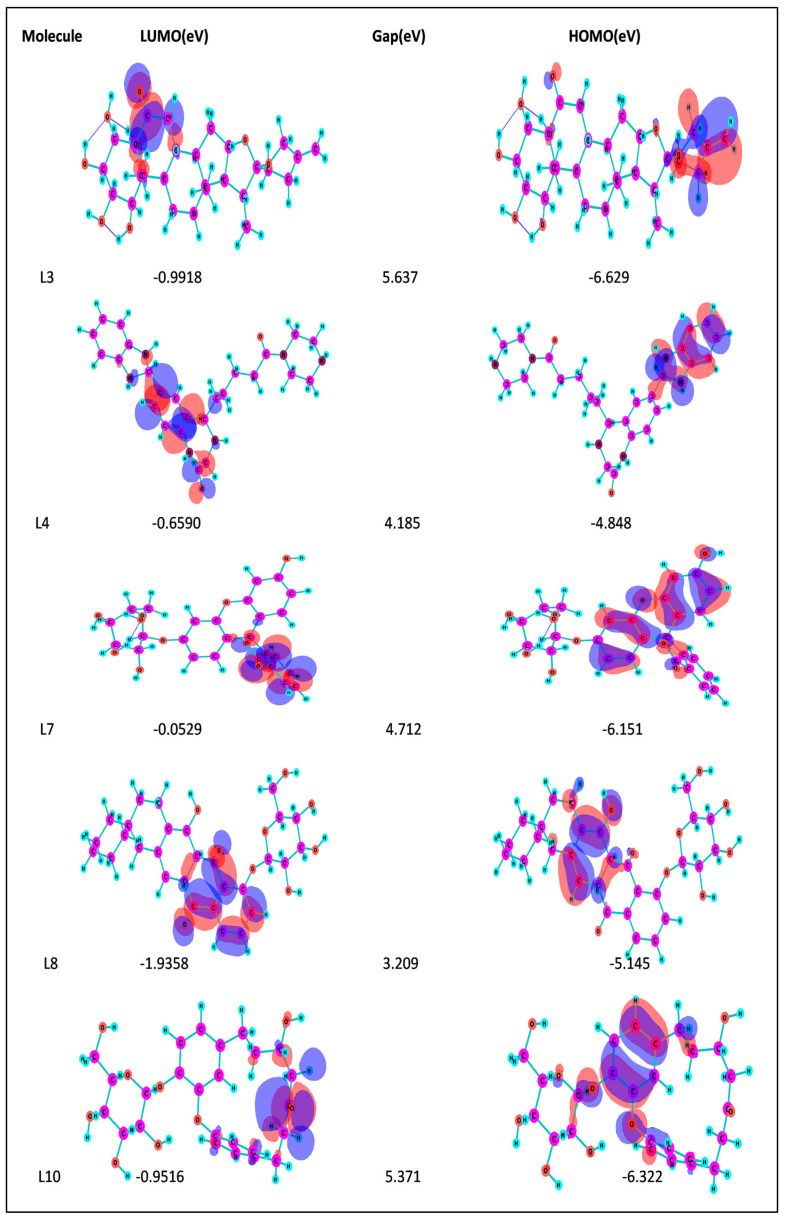
Molecular orbitals showing HOMO-LUMO and energy gap of the selected ligands.

Figure 4 represents the HOMO-LUMO orbitals of the selected five compounds with energy gap values. The computed FMO energy band gap values, presented in Table 12, were notably high, signifying elevated kinetic stability and reduced chemical reactivity of the molecules. The energy gap for ligand L3 is comparatively higher and considered to be more stable, whereas ligand L8 has a lower energy gap and is considered as less stable. The energy gap between the HOMO and LUMO is very high for all the selected molecules, indicating their stability and lower reactivity, due to which the geometry optimized structure does not show considerable difference in binding affinity during molecular docking between the initial and optimized structure.

### 2.11. Redocking and Interaction Analysis

The redocking process was executed, involving the envelope protein and the ligands that had undergone geometric optimization, employing AutoDock 4.0. Upon optimization, ligands L3, L4, and L10 exhibited improved binding energy compared with their initial values, as indicated in Table 13. In contrast, the binding energy of ligands L7 and L8 decreased after optimization. This suggests that the quantum-mechanics-based (QM-based) optimization of these ligands had a positive impact on their binding characteristics. To gain deeper insights, a comprehensive analysis of protein–ligand interactions was conducted to identify the specific amino acid residues involved in the binding. Understanding these interactions plays a pivotal role in the development of drugs and the treatment of disease. Therefore, the interactions between the envelope protein and ligands were thoroughly examined using BIOVIA Discovery Studio Visualizer.

**Figure 5:**
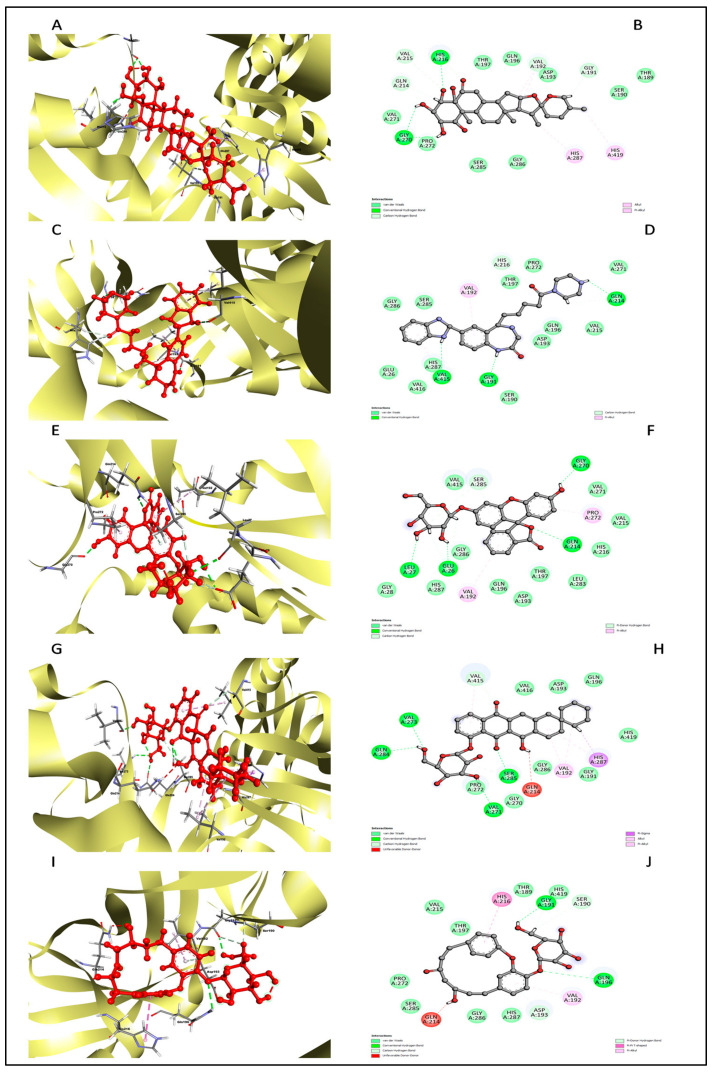
3D interaction between the envelope protein (yellow ribbon model) with the ligand (red ball & stick model) (**A**,**C**,**E**,**G**,**I**) and 2D interaction plot between envelope protein and different ligand (ball & stick model) (**B**,**D**,**F**,**H**,**J**).

The analysis of interactions between proteins and ligands holds a crucial role in the drug development process, aiding in the identification of lead molecules for combating various diseases. Consequently, we conducted an in-depth examination of the interactions between the protein and the ligand chosen using the BIOVIA Discovery Studio Visualizer tool. Table 14 and Figure 5 present the results of this interaction analysis, encompassing hydrogen bonding interactions (conventional, pi–donor H–B, and carbon H–B), various hydrophobic interactions (pi–alkyl, alkyl, pi–sigma, and pi–pi T-shaped), and electrostatic interactions (pi–anion).

The interaction analysis between the protein and L3 complex (Figure 5A,B) revealed a total of 11 bonding interactions, encompassing 7 hydrogen bonds (comprising 3 conventional and 4 carbon H–B bonds) and 4 hydrophobic interactions (including 2 alkyl and 2 pi–alkyl interactions). These interactions were associated with a binding energy of −7.58 kcal/mol. Notably, amino acid residues like His216 and Gly270 formed HN–O and H–O conventional hydrogen bonds with the ligand, while Val192 and 215, His216, and Gln214 engaged in carbon–hydrogen bonding with the ligand. Val192 established an alkyl interaction, whereas His287 and 419 contributed to pi–alkyl interactions with the ligand. In the case of the protein’s interaction with the L4 complex (Figure 5C,D), a total of seven bonding interactions were observed. These included five hydrogen bonds (comprising three conventional and two carbon H–B bonds) and two hydrophobic interactions (pi–alkyl interactions). The binding energy for this interaction was −8.91 kcal/mol. Residues Gly191 and 214, and Val415, participated in O–H conventional hydrogen bonding with the ligand, while His216 formed an HA–O carbon–hydrogen bond. Val192 and 415 contributed to pi–alkyl interactions with the ligand. Analysis of the protein’s interaction with the L7 complex (Figure 5E,F) revealed a total of seven bonding interactions, including five hydrogen bonds (comprising four conventional and one pi H–B bond) and two pi–alkyl hydrophobic interactions. This interaction possessed a binding energy of −6.43 kcal/mol. Amino acid residues such as Gly214, Glu26, Leu27, and Gly270 engaged in conventional hydrogen bonding with the ligand. At the same time, Ser285 formed a pi donor hydrogen bond, and Val192 and Pro272 established pi–alkyl interactions with the ligand. On a similar note, the interaction analysis of the protein with the L8 complex (Figure 5G,H) revealed a total of 13 bonding interactions. These included eight hydrogen bonds (comprising six conventional and two carbon H–B bonds) and five hydrophobic interactions (including one p–sigma, two alkyl, and two pi–alkyl interactions). This interaction exhibited a binding energy of −6.12 kcal/mol. Residues such as Val273, Ser285, Gln284, and Val271 participated in conventional hydrogen bonding with the ligand, while His287, Val192, His287, and Val415 contributed to hydrophobic interactions. Furthermore, the interaction analysis of the protein with the L10 ligand (Figure 5I,J) involved five bonding interactions (two conventional and one pi–donor hydrogen bond) and two hydrophobic interactions (one pi–pi T-shaped and one pi–alkyl). Amino acid residues like Gln196, Gly191, Asp193, His216, and Val192 were involved in these interactions. Based on this interaction analysis, ligands L3 and L4 were chosen for subsequent simulation studies.

### 2.12. Molecular Dynamics (MD) Simulation Studies

#### 2.12.1. Simulation of Envelope Protein

**Figure 6:**
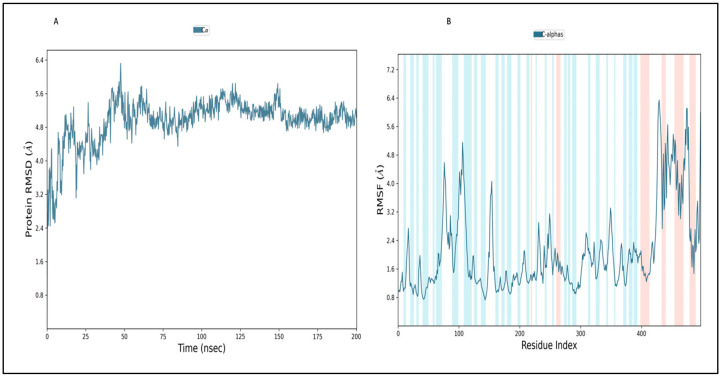
MD simulation analysis of 200 ns trajectories for the envelope protein. RMSD of the Cα backbone of the envelope protein is represented in (**A**), and RMSF of the Cα backbone of the envelope protein is represented in (**B**).

In order to evaluate the stability and convergence of the envelope protein, MD simulations were conducted over 200 ns duration. The root-mean-square deviation (RMSD) trajectory of the envelope protein initially began at 3.2 Å at 0 ns and increased gradually to 5.2 Å at 25 ns. It reached a maximum RMSD of 6.2 Å at 50 ns before stabilizing around 5.2 Å (Figure 6A). This range of RMSD values falls within an acceptable range. Substantial deviations could indicate significant conformational changes in the protein during the MD simulation, which were observed in the initial 50 ns of simulation time. Furthermore, convergence of the simulation and stabilization of the RMSD values around a fixed value are of the utmost importance. A stable RMSD plot throughout the simulation signifies good convergence and stable protein conformations, which were observed after 100 ns simulation time. The root-mean-square fluctuations (RMSF) plot revealed notable fluctuations in the envelope protein, primarily observed in C-terminal residues from 420 aa to 490 aa, possibly indicating higher flexibility in these regions (Figure 6B). Consequently, the RMSF plots suggest that the protein structure remains rigid during the simulation. Secondary structural elements, such as alpha-helical regions (depicted in red) and beta-stranded areas (in blue), are also annotated in the RMSF plot and were found to be stable and showed fewer fluctuations during simulation. The other secondary structural elements, like random coil, accounted for 40% of the amino acid as depicted by SOPMA and showed higher fluctuation during simulation.

#### 2.12.2. Simulation of L3 Complex

**Figure 7:**
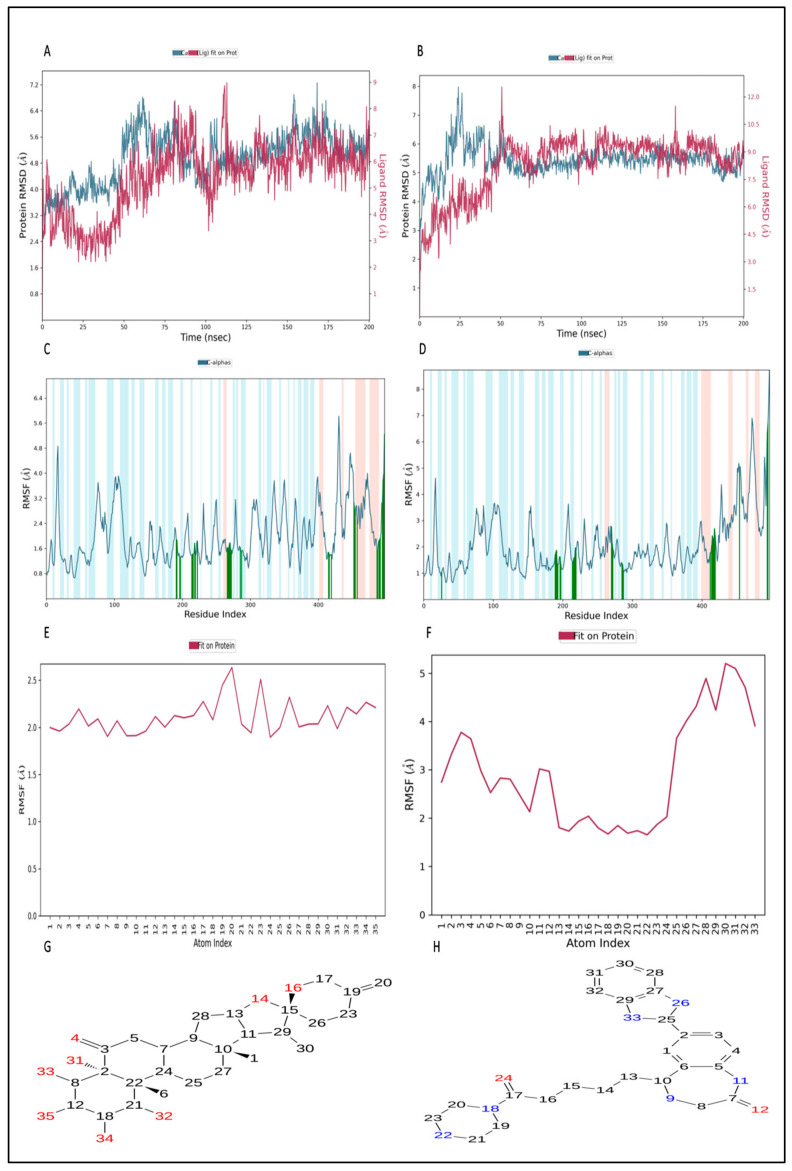
RMSD trajectory, RMSF plot, ligand RMSF plot, and ligand structure of the envelope protein–L3 complex (**A**,**C**,**E**,**G**) and envelope protein–L4 complex (**B**,**D**,**F**,**H**).

Figure 7A provides a visual representation of the RMSD trajectory for the envelope protein–L3 complex. The provided picture illustrates the trajectory of the protein’s RMSD in blue, with the corresponding RMSD values presented in Å units on the *Y*-axis (left). Additionally, the figure displays the trajectory of the ligand’s RMSD in red, with the corresponding values also in Å units on the *Y*-axis (right). The RMSD of the protein displayed fluctuations beginning around 2.80 Å at 0 ns and gradually increasing to above 6.8 Å by 60 ns. Subsequently, the protein’s RMSD trajectory decreased and fluctuated between 5.8 Å and 6.4 Å between 60 ns and 150 ns. After 150 ns, it increased to 6.8 Å and 7.2 Å at 175 ns. Further, the protein RMSD stabilized around 5.44 Å during the end of the 200 ns MD simulation. The protein’s RMSD exhibited fluctuations primarily within the range of 2.8 Å to 5.8 Å, with some intermediate spikes around 7.2 Å, suggesting that the protein maintained a stable conformation throughout the simulation. Contrastingly, the ligand’s RMSD trajectory initiated around 3.16 Å but sharply increased, reaching approximately 6 Å at the initial stage of 5 ns. After 5 ns, the RMSD of the ligand fluctuated around 3.0 Å up to 50 ns. From 50 ns to 120 ns, the ligand RMSD fluctuated between 5.8 Å and 9.0 Å. From 120 ns onwards, the ligand’s RMSD exhibited fluctuations around 5.8 Å, suggesting a stable conformation at the end of the simulation time. In general, the RMSD of the ligand suggests the ligand stayed stable at the binding pocket of the protein and interacted with different amino acids to reach stable binding at the end of the simulation time.

**Figure 8:**
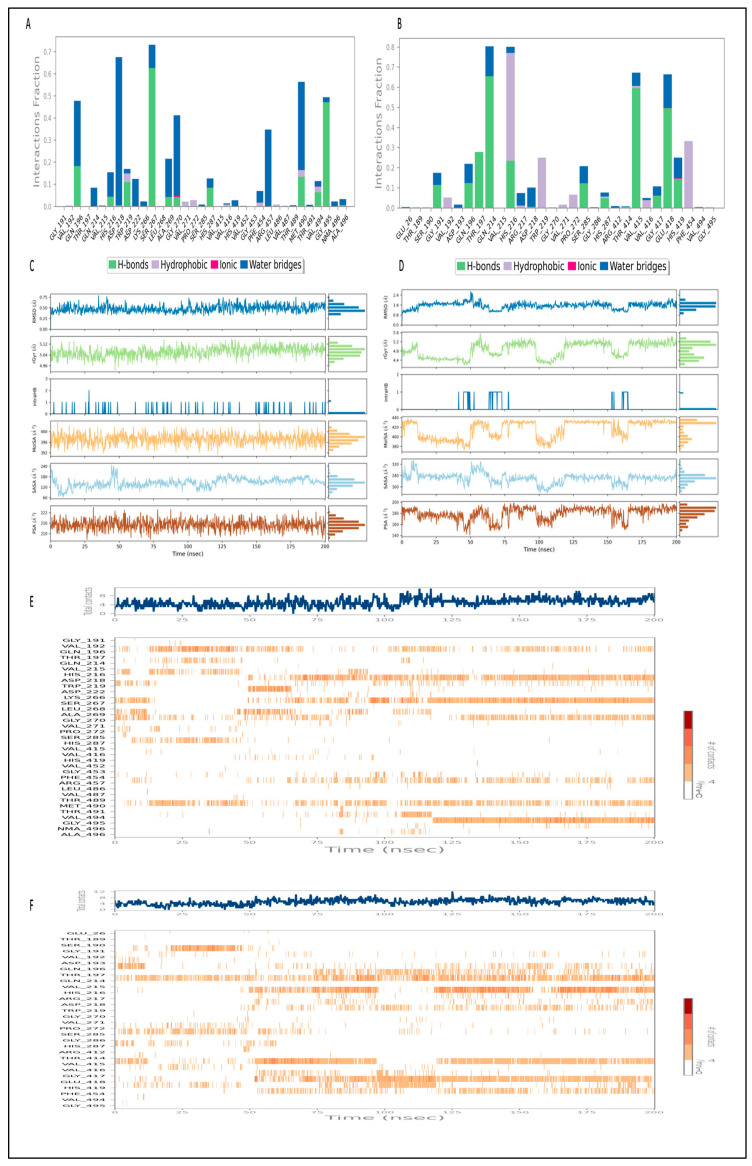
Protein–ligand interaction diagram, ligand properties, and interaction timeline of envelope protein–L3 complex (**A**,**C**,**E**) and envelope protein–L4 complex (**B**,**D**,**F**).

Turning to the RMSF plot of the protein in the envelope protein–L3 complex (Figure 7C), the bars in the green color seen in the plot correspond to instances where amino acids are engaging in interactions with the ligand. While amino acids at the C-terminal displayed higher RMSF values, the average RMSF for most amino acids remained under 2.0 Å. RMSF values for the majority of the interacting amino acid residues were below 2.0, with the exception of a few C-terminal amino acids. It is noteworthy that the amino acids at the terminal exhibited higher RMSF values, varying from 4.0 to 5.6 Å. This observation was particularly pronounced in the C-terminal region, characterized by a loop conformation, leading to significant RMSF fluctuations [60]. In a side-by-side comparison with the protein’s free state (as depicted in Figure 6B), the envelope protein–L3 complex exhibited lower RMSF values, which are likely a result of the binding of the L3 ligand in the loop region of the protein. This suggests that the complex experiences lower RMSF fluctuations compared with the protein in its free state. It is worth noting that secondary structure elements, such as alpha helices and beta strands, typically exhibit less fluctuation compared with loop regions (indicated by a white background). The ligand’s RMSF plot for L3 in the envelope protein–L3 complex is presented in Figure 8E. The RMSF diagram of the ligand, on average, reveals that every atom of L3 possesses an RMSF value between 2.0 and 2.8 Å. This suggests that the ligand was stable at the binding pocket of the envelope protein but explores binding to different amino acids within the binding pocket to reach a more stable configuration.

**Figure 9:**
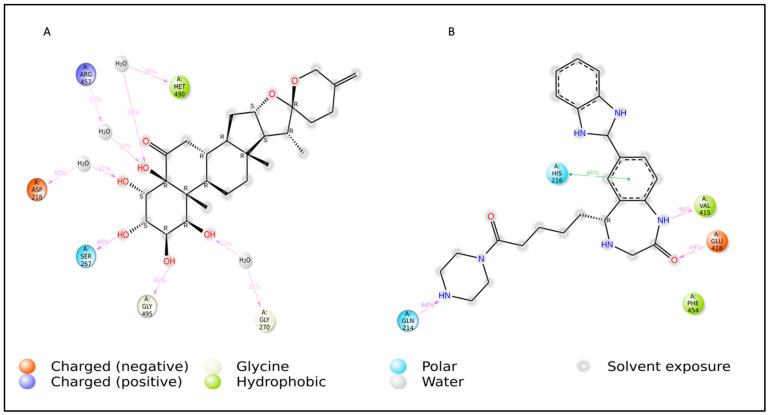
Ligand–protein contact for more than 30% of simulation time; (**A**) L3–envelope protein complex; (**B**) L3–envelope protein complex.

Four subtypes of protein–ligand contacts are distinguished in Figure 8A for the envelope protein–L3 complex during the 200 ns MD simulation: hydrophobic, ionic, hydrogen bonds, and water bridges. Throughout the trajectory, these layered bar charts are normalized; greater interaction fractions indicate more sustained interactions. L3 established hydrogen bonds (conventional) between amino acids and ligands during the MD simulation, including Gln196, Gln214, His216, Asp218, Trp219, Asp222, Lys266, Ser267, Ala269, Gly270, His287, Arg457, Met490, Val494, and Gly495. A summary of the interactions and contacts (including hydrophobic, ionic, H-bonds, and water bridges) is illustrated in Figure 7E as a timeline. Figure 9A shows the ligand interaction with the envelope protein with more than 30% of simulation time. The amino acids Asp218, Ser267, Gly270, Arg457, Met490, and Gly495 maintained 42%, 49%, 32%, 32%, 38%, and 40% of contact time, respectively, during the simulation. Throughout the trajectory, the total number of specific contacts between the protein and the ligand is displayed in the top panel. Figure 7E provides insight into the MD simulation, suggesting that the ligand changed its initial binding site amino acids during the first 75 ns. However, after 75 ns, L3 exhibited a change in the pattern of ligand contacts and maintained it throughout 200 ns. This aligns with the observation in Figure 7A, indicating fluctuations in the ligand’s RMSD and, in turn, an unstable binding pose of the ligand. This representation allows for a dynamic view of the evolving interactions between the protein and the ligand throughout the 200 ns MD simulation.

Finally, Figure 8C presents various properties of L3 in the envelope protein–L3 complex, including the RMSD, radius of gyration (rGyr), molecular surface area (MolSA), intramolecular hydrogen bond, polar surface area (PSA), and solvent-accessible surface area (SASA). The ligand’s RMSD fluctuated between 0.3 Å and 0.75 Å, stabilizing at around 0.55 Å after 175 ns. The rGyr fluctuated between 4.94 Å and 5.14 Å, reaching equilibrium at approximately 5.10 Å after 150 ns. There were only two intramolecular hydrogen bonds within the L3 molecule. The MolSA fluctuated between 392 Å^2^ and 404 Å^2^, maintaining equilibrium around 400 Å^2^. The SASA of L3 fluctuated between 60 Å^2^ and 240 Å^2^, stabilizing at around 180 Å^2^ after 150 ns. The PSA of L3 fluctuated between 206 Å^2^ and 226 Å^2^, stabilizing at around 216 Å^2^. These properties collectively provide insights into the dynamic behavior and structural changes experienced by the L3 ligand during the simulation.

#### 2.12.3. Simulation of L4 Complex

Figure 7B provides a visual representation of the RMSD trajectory for the envelope protein–L4 complex. The provided picture illustrates the trajectory of the protein’s RMSD in blue, with the corresponding RMSD values presented in Å units on the *y*-axis (left). Additionally, the figure displays the trajectory of the ligand’s RMSD in red, with the corresponding values also in Å units on the *y*-axis (right). The protein’s RMSD displayed fluctuations, beginning around 2.45 Å at 0 ns and gradually increasing to above 7.9 Å by 25 ns. Subsequently, the protein’s RMSD trajectory decreased to approximately 7.0 Å around 50 ns. From 50 ns onward, the RMSD trajectory exhibited decreasing fluctuations, ultimately stabilizing around 5.8 Å until the end of the 200 ns MD simulation. The protein’s RMSD predominantly exhibited a range of 2.4 Å to 5.8 Å, with a small intermediate spike of 7.9 Å at 25 ns, which suggests that the protein remained stable during the MD simulation. Contrastingly, the ligand’s RMSD trajectory initiated around 2.34 Å at 0 ns and increased up to 12.53 Å at 50 ns. After 50 ns, the RMSD of the ligand fluctuated between 7.0 Å and 10 Å. From 100 ns onwards, the ligand’s RMSD exhibited fluctuations around 8.7 Å, suggesting the stable conformation at the end of the simulation time. In general, the RMSD of the ligand suggests the ligand stayed stable at the binding pocket of the protein and interacted with different amino acids to reach the stable binding position at the end of the simulation time.

The protein’s RMSF plot in the envelope protein–L4 complex (Figure 7D) illustrates the interaction between the ligand and amino acids, as indicated by the bars in green in the plot. While C-terminal amino acid residues displayed higher RMSF values, the average RMSF for most amino acids remained under 3.0 Å. Many interacting amino acids showed RMSF values lower than 2.0 Å, except for a few C-terminal amino acids. In a side-by-side comparison with the protein’s free state (as depicted in Figure 6B), the envelope protein–L4 complex exhibited higher RMSF values, which are likely a result of the binding of the L4 ligand in the loop region of the protein. This suggests that the complex experiences more significant RMSF fluctuations compared with the protein in its free state. The protein RMSF plot emphasizes the stability of individual amino acid residues during the 200 ns MD simulation. It is worth noting that secondary structure elements such as alpha helices and beta strands typically exhibit less fluctuation compared with loop regions (indicated by a white background). The ligand’s RMSF plot for L4 in the envelope protein–L4 complex is presented in Figure 7F. The RMSF diagram of the ligand, on average, reveals that every atom of L4 possesses an RMSF value in the range of 1.8 to 5 Å. This suggests that the ligand continuously explores different binding poses in search of a more stable configuration in the binding pocket of the envelope protein.

During the 200 ns MD simulation, the protein–ligand contacts for the envelope protein–L4 complex are classified into four subtypes in Figure 8B: hydrophobic, ionic, hydrogen bond, and water bridge. Throughout the trajectory, these layered bar charts are normalized; greater interaction fractions indicate more sustained interactions. During the MD simulation, L4 formed conventional hydrogen bonds with amino acids including Gly191, Gln196, Thr197, Gln214, His287, Val415, Gly417, Glu418, and His419. A summary of the timeline depicting the interactions and contacts (including hydrophobic, ionic, H-bonds, and water bridges) can be found in Figure 8D. Figure 9B shows the ligand interaction with the envelope protein with more than 30% of simulation time. The amino acids Gln214, His216, Val415, and Glu418 maintained 64%, 46%, 56%, and 49% of contact time, respectively, during the simulation. Throughout the trajectory, the total number of specific contacts between the protein and the ligand is displayed in the upper panel. Figure 8F provides insight into the first 50 ns of the MD simulation, suggesting that the ligand changed the interaction with the amino acids within the defined binding pocket. However, after 50 ns, L4 exhibited a constant binding pattern of ligand contacts. This aligns with the observation in Figure 7B, indicating fluctuations in the ligand’s RMSD and, in turn, an unstable binding pose of the ligand in the initial 50 ns of the simulation run.

Finally, Figure 8D presents various properties of L4 in the envelope protein–L4 complex, including RMSD, rGyr, MolSA, intramolecular hydrogen bond, PSA, and SASA. The ligand’s RMSD fluctuated between 0.8 Å and 2.4 Å, stabilizing at around 1.6 Å after 75 ns. The rGyr fluctuated between 4.4 Å and 5.6 Å, reaching equilibrium at approximately 5.3 Å after 160 ns. There was only one intramolecular hydrogen bond within the L4 molecule. The MolSA fluctuated between 380 Å^2^ and 440 Å^2^, maintaining equilibrium around 430 Å^2^ after 160 ns. The SASA of L4 fluctuated between 150 Å^2^ and 320 Å^2^, stabilizing at around 240 Å^2^ after 160 ns. The PSA of L4 fluctuated between 140 Å^2^ and 200 Å^2^, stabilizing at around 190 Å^2^ after 160 ns.

**Figure 10:**
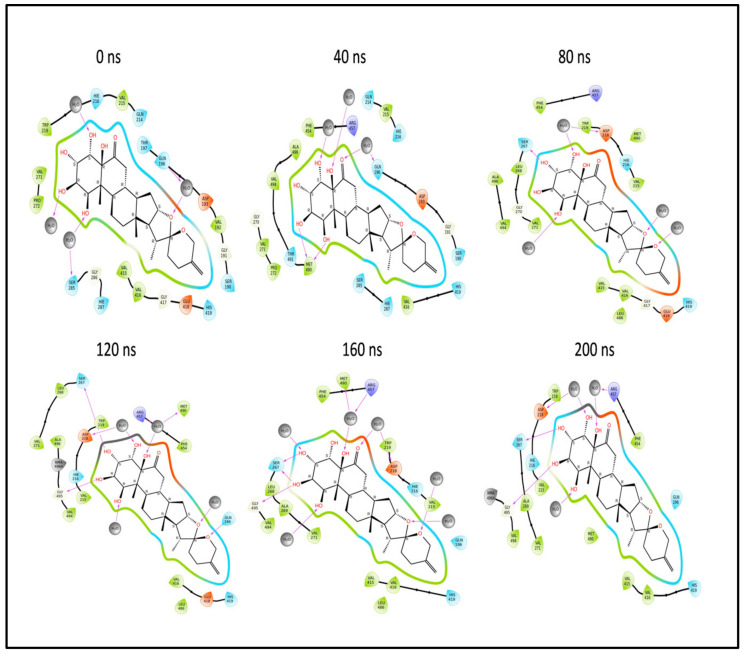
Binding poses of L3 ligand at the active site of the envelope protein at different time intervals during MD simulation.

**Figure 11:**
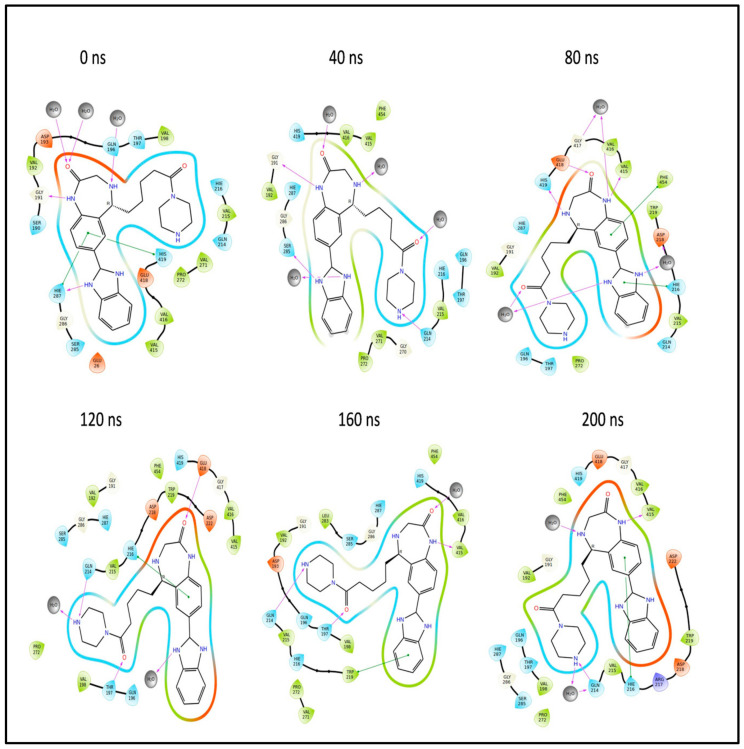
Binding poses of L4 ligand at the active site of the envelope protein at different time intervals during MD simulation.

In summary, during the 200 ns MD simulation, both L3 and L4 ligands underwent conformational changes at the active binding pocket as they attempted to stabilize themselves by finding more suitable binding residues within the defined binding site. This is evident in the ligand RMSD trajectories presented in Figure 7A,B. Additionally, as the ligands sought to stabilize themselves, they interacted with different amino acid residues over time, as shown in Figure 8A,B. The various binding poses of L3 and L4 at the active binding pocket of the envelope protein at different time points, including 0 ns, 40 ns, 80 ns, 120 ns, 160 ns, and 200 ns, are depicted in Figure 10 and Figure 11, respectively.

### 2.13. Binding Free Energy Analysis of the L3 and L4 Complex

In this study, we employed the Prime MM-GBSA approach to estimate the binding free energies of ligand–protein complexes in MD simulations. We considered various energy components, including electrostatic interactions, covalent contributions, hydrogen bonding, van der Waals forces, self-contact energies, lipophilic interactions, and solvation effects. These components were collectively added, adhering to the principle of additivity, to calculate the binding free energy (ΔGbind) expressed in kilocalories per mole (kcal/mol). The ΔGbind values provide insights into the strength and favorability of ligand–protein interactions. This method is commonly used in drug discovery and molecular dynamics research.

Using the MD simulation trajectory, we computed the binding free energy and other contributing energies in the form of MM-GBSA for the L3 and L4 complexes. The results, as shown in Table 15, indicate that the average binding free energy of the envelope protein with the L3 complex (−85.26 ± 4.63 kcal/mol) is higher than that of the L4 complex (−66.60 ± 2.92 kcal/mol) by a margin of −18.66 kcal/mol. This suggests that the envelope protein–L3 complex exhibits higher stability compared with the L4 complex. From the total energy, the primary contribution was made by van der Waals forces: for the L3 complex, the ΔG_bind_vdW is −70.63 ± 3.57 kcal/mol, and for the L4 complex, it is −53.79 ± 1.23 kcal/mol. Further coulombic interactions give further stability to the protein–ligand complex. The ΔG_bind_Coulomb for the L3 and L4 complexes is −43.66 ± 4.48 and−23.60 ± 1.52 kcal/mol, respectively.

## 3. Discussion

In light of the vaccine’s limited efficacy in combating KFDV, the public health response becomes more susceptible and less capable of effectively addressing the situation [15,27,61,62]. The increasing number of KFDV cases and the expansion of endemic areas in India necessitate an interdisciplinary approach to the development of antiviral drug candidates for KFDV [4,63]. Therefore, the present study aimed to inhibit the function of the envelope protein interaction of KFDV with host cells [64,65,66] by identifying a novel and effective antiviral drug candidate against KFDV by a de novo design and pharmacophore-based screening approach.

In this study, we retrieved the envelope protein sequence from the UniProt database with UniProt ID D7RF80.1 and performed sequence analysis. MSA revealed the conservation of 12 cysteine residues to form disulfide bonds between 3–30, 60–121, 74–105, 92–116, 186–290, and 307–338 amino acids among all flaviviruses including dengue virus, suggesting similar 3D structures [29]. Due to the unavailability of the 3D structure of the envelope protein, the 3D structure was determined by Robetta and I-Tasser servers. Based on the structure validation method, the model generated from Robetta was selected and further subjected to energy minimization using the YASARA energy minimization server. Previous studies also reported that the Robetta server provides reliable 3D structure prediction [41,67]. The refined 3D structure of the envelope protein showed a drastic change in ERRAT score from 89.85 to 94.19, which represents that the refined model is of good quality [68,69] and is further considered for drug designing. The active site of amino acids Ile^48^, His^49^, Gln^50^, Pro^194^, Val^215^, Val^273^, Ala^274^, and Gly^286^ with coordinates 15.707, 0.627, and 11.566 of the envelope protein was determined by the COACH-D server; it also predicted the tentative ligand binding at the BOG pocket based on the PDB template 1OEK.B of the dengue virus envelope protein. A similar BOG pocket was identified in the crystallographic structure of the dengue virus envelope protein bound by the inhibitor BOG between the domains 1 and 2 [29,70]. This pocket plays a vital role in conformational changes between immature and mature flavivirus, including domain orientation of the envelope protein. This suggests the binging of ligands at the BOG pocket could inhibit a viral life cycle, leading to the development of antiviral compounds [71,72,73,74]. By considering this, BOG was used as a template for ligand design by different approaches. Three methods, de novo designing by eLea3D, pharmacophore screening by Pharmit, and ligand screening by PubChem database generated 50, 9243 (7374 from the COCONUT database and 1869 from the Zinc database), and 3808 compounds, respectively. Out of all the identified compounds, virtual screening of 146 compounds (15 from de novo designing, 98 from pharmacophore screening, and 33 from ligand screening) by PyRx was carried out. Based on the binding energy value, 12 compounds were selected. The 12 chosen compounds were subjected to toxicity analysis using ProTox II, and compounds 16178612 (L1), CNP0247967 (L3), SA8(L4), ZINC0001000052673(L7), CNP0097629.2(L8), and CNP0247704.2 (L10) were chosen for further analysis in the context of ADME by the SwissADME server. The compound L3 showed mild cytotoxicity and good oral availability, with an LD50 of 10,000 belonging to class 6. The L3 drug can be prepared with a concentration that can induce antiviral activity without inducing cytotoxicity [75]. Based on the ADME property, five compounds, namely L3, L4, L7, L8, and L10, were selected for geometry optimization. Geometry optimization of the chosen compounds was carried out by DFT based on Orca 4.2.1. The FMO-based HOMO-LUMO energy gap was also calculated to evaluate the chemical reactivity of the compounds. The energy gap for all the compounds was higher than 3.50 eV, indicating the high kinetic stability and low reactivity corresponding to the bioactivity of the compounds [76]. The geometry-optimized compounds were redocked using AutoDock 4.0 with an envelope protein, which decreased the docking energy of compound CNP0247967 (L3) from −7.26 to −7.58 kcal/mol, that of SA8 (L4) from −7.82 to −8.91 kcal/mol, and for the remaining compounds showed an increase in binding affinity [77,78].

Molecular docking must be validated with MD simulation for at least 100 ns to find the stability of the protein–ligand complex for in silico analysis [79,80,81]. The geometry-optimized redocking complex structure for the envelope protein–L3 and L4 complex underwent MD simulation to assess the stability of the compounds within the protein’s binding site. The 200 ns simulation trajectories, including RMSD, RMSF, protein–ligand contact mapping, and ligand properties [79], were analyzed using Desmond 2022.4 version software. In the current study, both the envelope protein–L3 and envelope protein–L4 complexes exhibited stable conformations. However, ligands L3 and L4 in the protein–ligand complexes attempted to explore different binding poses within the binding pocket while maintaining stability during the 200 ns simulation. Similar results, where ligands sought stable binding sites during MD simulation, have been reported [79,82,83].

Furthermore, an analysis of the RMSF for both the protein and the ligand was conducted through an all-atom MD simulation. Terminal amino acids generally exhibited higher RMSF values compared with other residues, with the ideal values being lower than 2 Å. Amino acid residues interacting with the protein should also display RMSF values below 2 Å. Throughout the 200 ns MD simulation, both L3 and L4 ligands maintained significant interactions with the protein. Correlations between ligand RMSD, ligand RMSF, and protein–ligand contacts were observed. Various ligand properties, including RMSD, rGyr, MolSA, SASA, and PSA, were also thoroughly examined.

Furthermore, the MM-GBSA approach was employed to estimate the binding free energies of the ligand–protein complexes in MD simulation. The average binding free energy of the envelope protein with the L3 complex was found to be lower (−85.26 ± 4.63 kcal/mol) than that of the L4 complex (−66.60 ± 2.92 kcal/mol) by a margin of −18.66 kcal/mol. These findings suggest that the two ligands, L3 and L4, have the potential to act as inhibitors for the envelope protein. However, further validation through in vitro studies [84] is required to confirm their efficacy.

## 4. Materials and Methods

### 4.1. Materials

**Figure 12:**
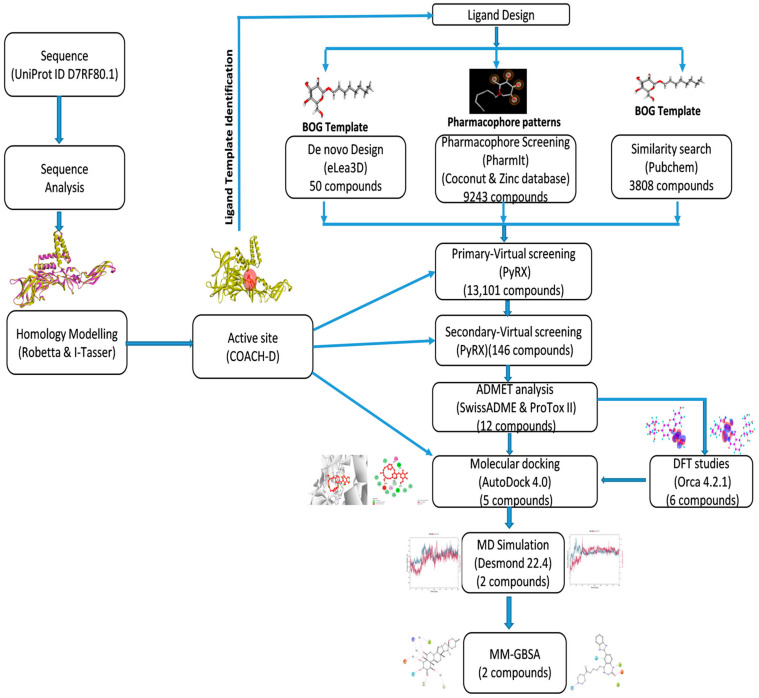
Methodology schema employed in the study for predicting inhibitors for the envelope protein of KFDV.

In this research, various resources were employed, including databases such as UniProt, PDB, PubChem, COCONUT, and the ZINC database. Additionally, a range of software programs were utilized to perform structural and functional analyses on the protein. These programs include BLASTp, Clustal Omega, Swiss Model, I-Tasser, Robetta, COACH-D, PyRx 0.8, AutoDock 4.0, ORCA 4.2.1, ChemCraft 1.8, SwissADME, Protox-II, Open Babel, Discovery Studio 2021, and Desmond 2022.4. A workflow schema detailing the stepwise techniques employed in this study is shown in Figure 12.

### 4.2. Methods

#### 4.2.1. Sequence Retrieval and Analysis

The envelope protein sequence was obtained from the UniProt database www.uniprot.org (accessed on 20 December 2023) in FASTA format [85]. To analyze the physicochemical properties of the chosen protein, the ProtParam tool https://web.expasy.org/protparam (accessed on 22 December 2023) on the ExPASy server was employed [86]. After obtaining the sequence, a search for homologous sequences was conducted using the BLASTp tool https://blast.ncbi.nlm.nih.gov/Blast.cgi (accessed on 30 December 2023), employing NCBI’s BLASTp algorithm [87] and utilizing the PDB as the database. The top five sequences, determined by their e-value and percentage similarity, from different viruses were selected for further analysis in multiple sequence alignment and phylogenetic analysis, which was performed using the Clustal Omega tool [88]. The secondary structure and transmembrane region of the envelope protein were determined by SOPMA [89] and TMHMM 2.0 [90], respectively.

#### 4.2.2. Homology Modeling and Structure Refinement

In the absence of a crystalline framework for the envelope protein, we predicted its 3D structure by leveraging the I-Tasser and Robetta servers. I-Tasser employs an iterative threading assembly simulation involving four primary stages: identification of threading templates, iterative simulation of structure assembly, selection and refinement of models, and annotation of structure-based functions [91]. The Robetta server http://robetta.bakerlab.org (accessed on 31 December 2023) dissects the submitted sequence into potential domains, creating structural models through either comparative modeling or de novo structure prediction methods [92]. To achieve the optimal conformation with the least energy for the constructed 3D envelope protein structure, we utilized the YASARA Energy Minimization server [93].

#### 4.2.3. Structure Validation

Several validation methods were employed to assess the accuracy of the predicted structure. The initial step involved estimating the overall quality of the protein using the ERRAT program [94]. Next, the stereochemical analysis of the protein was assessed through a Ramachandran plot generated by the PROCHECK server [95]. The third step entailed comparing the predicted model with existing experimental structures in the database, which was accomplished by evaluating the QMEAN score [96] and using ProSAweb [97]. The fourth and final step involved evaluating the compatibility between the 3D structure and the amino acids, a task carried out using VERIFY 3D [98].

#### 4.2.4. Binding Site/Active Site Prediction

The active site of the predicted model was determined using COACH-D. COACH-D employs an algorithm that predicts ligand binding sites by referencing the BioLip database. It does so through comparisons of binding-specific substructures and sequence profiles. The results from COACH-D regarding binding sites are then ranked based on their confidence score, known as the c-Score [99].

#### 4.2.5. Ligand Design

Ligand design is a critical approach in computational drug design, focusing on the identification of potential small molecules that can inhibit a specific protein. In the present study, two distinct approaches were employed for ligand design: the structure-based approach and the ligand-based approach. In the ligand-based approach, Pharmit and database screening were utilized, while in the structure-based approach, eLea3D was employed**.** Analogs of Octyl beta-D-glucopyranoside (BOG), with PubChem ID 62,852, was utilized to design ligands by different approaches targeting the BOG pocket of the envelope protein. A similar approach was carried out for defining the antivirals targeting the BOG pocket of the envelope protein [100,101].

##### De Novo Design

A drug targeting the active site of the envelope protein was developed using the e-LEA3D server, which incorporates the PLANTS docking program. During the drug design process, molecules were designed while adhering to Lipinski’s Rule of Five as a constraint. This involved the implementation of a genetic algorithm with an initial population consisting of FDA-approved drugs and a maximum population size of 50 molecules [102].

##### Pharmacophore Screening

The Pharmit server was employed to screen ligands from the COCONUT and Zinc databases based on pharmacophore patterns generated by the Webina server. The pharmacophore model was developed using the Pharmit server http://pharmit.csb.pitt.edu (accessed on 16 January 2024). The pharmacophore features were derived from the COACH-D complex of the envelope protein with the Octyl beta-D-glucopyranoside inhibitor. Subsequently, the COCONUT database (comprising 8,739,148 conformers) and the Zinc database (comprising 122,276,899 conformers) were searched using this model. The hit compounds’ poses were then optimized using Pharmit’s functions. Finally, the pharmacophore-based, optimized entries were subjected to further docking with the target protein to identify lead compounds with the best docking scores [103].

##### Ligand-Based Screening

The ligand identified by the active site prediction server, Octyl beta-D-glucopyranoside, with PubChem ID 62852, was used as a template to search for similar ligands based on Lipinski’s rule from the PubChem database [104]. The entire PubChem database was curated using several criteria, which included molecular weight (MW) within the range of 160 to 560; hydrogen bond (HB) acceptors and donors not exceeding 10 and 5, respectively; the number of rotatable bonds in the range of 0 to 21; the number of atoms not exceeding 40; and hydrophobicity (log P) less than or equal to 5.

#### 4.2.6. Virtual Screening

Virtual screening (VS) proves indispensable in identifying potential inhibitors for the envelope protein from a vast array of ligands from the database. All drug-like compounds resulting from various ligand design methods were amalgamated into a unified SDF file. This SDF file was then brought into Open Babel within PyRx 0.8 [105], where the ligands underwent further conversion into AutoDock PDBQT format. The virtual screening was conducted in PyRx using the AutoDock Vina [44] option, employing an “exhaustiveness” setting of 8. For defining the Vina search space, a grid box centered at x 15.707, y 0.627, z 11.566, with dimensions of 34.4047 Å in the *x*-axis, 23.6217 Å in the *y*-axis, and 38.8008 Å in the *z*-axis, was established. This grid was designed to encompass the active site residues identified by COACH-D. The best pose from each ligand design approach was selected and ranked based on their binding affinities.

#### 4.2.7. ADMET Studies

Assessing drug-likeness and ADME-T (absorption, distribution, metabolism, and excretion toxicity) properties is vital in drug discovery. The canonical SMILES of each molecule served as the input for ADME predictions through the SwissADME server [106]. Furthermore, the ProTox II web server [107] predicted various toxicity endpoints, including hepatotoxicity, carcinogenicity, oral toxicity, mutagenicity, and cytotoxicity. The chemicals were categorized into six toxicity classes (1–6) by ProTox II, where class 1 represents the highest level of risk (LD50 < 5), while class 6 denotes compounds that are non-toxic (LD50 > 5000). The top 12 compounds with the most favorable binding energies from the PyRx results were selected to conduct subsequent ADMET investigations.

#### 4.2.8. Geometry Optimization of the Selected Ligand Compounds

Geometric optimization calculations were conducted using the density functional theory (DFT) method. The quantum-mechanical wave function contains comprehensive information about the system under investigation. Optimization and vibration frequency calculations were carried out using Becke’s three parameters with the Lee–Yang–Parr functional (B3LYP) level of theory with the ORCA version 4.2.1 program [108]. B3LYP is a hybrid density functional theory method that combines different types of functionals. To facilitate these calculations, ORCA input files were generated using AVOGADRO version 1.2.0 software [109]. Frequency calculations were performed to obtain thermodynamic properties and to confirm that each optimization step led to an energy minimum, ensuring the stability of the calculated molecular structures.

#### 4.2.9. Frontier Molecular Orbital HOMO/LUMO Calculation

Within electrophilic–nucleophilic processes, the movement of electrons transpires from the HOMO to the LUMO, establishing an energy differential known as the HOMO-LUMO gap. This gap holds considerable significance in organic chemistry, furnishing valuable perspectives into the photochemistry, potency, and resilience of organic transition metal compounds. To gauge the susceptibility of atoms to electrophilic and nucleophilic interactions, the HOMO and LUMO energies were computed and visually represented using the ChemCraft tool [110]. The energy difference between these two orbitals, designated as the HOMO-LUMO gap, was ascertained by employing a specific equation. This computation offers insightful information regarding the reactivity and chemical characteristics of the scrutinized molecules.

#### 4.2.10. Molecular Docking

Molecular docking is a critical technique in computational drug design, assessing ligand interactions with a protein’s active site, including binding energy, free energy, and complex stability. Ligands chosen after ADMET screening underwent geometry optimization and were docked with the envelope protein using the MGL 1.5.7 tool, employing AutoDock 4.0 [111,112]. In docking simulations, polar hydrogen atoms and Kollman charges were added to the receptor and ligands, respectively. The grid for docking consisted of 60 points in each dimension, with dimensions x 15.707 Å, y 0.627 Å, and z 11.566 Å. Default parameters of Auto Grid 4.0 calculated affinity maps, desolvation maps, and electrostatic maps. The Lamarckian genetic algorithm (LGA) explored ligand conformations, running for 50 sets with a maximum of 25,000,000 energy evaluations per run and a maximum of 27,000 generations. Mutation and crossover rates were set at 0.02 and 0.8, respectively, with a crossover mode of 2. The Cauchy distribution’s mean and variance for gene mutation were 0 and 1, respectively. The number of generations for selecting the worst individual was set to 10. Docking was performed before and after geometric optimization to observe ligand binding effects on protein affinity. BIOVIA Discovery Studio 2021 software (BIOVIA, San Diego, CA, USA) [113] elucidated binding interactions between the envelope protein and ligands, aiding in understanding the protein–ligand interactions and binding modes.

#### 4.2.11. Molecular Simulation Studies for Protein and Complex

Desmond 2022.4 was utilized to conduct MD simulation experiments on the envelope protein and the envelope protein in complex with L3 and L4 [114]. At a temperature of 27 °C, separate simulations were carried out utilizing the OPLS-2005 force field [115,116,117] and an explicit solvent model comprising SPC water molecules contained within a periodic boundary salvation box of 10 Å × 10 Å × 10 Å [118]. To achieve a charge of 0.15 M neutralization, Na+ ions were introduced, accompanied by NaCl solutions, to simulate a physiological environment. To commence, the system was equilibrated for 10 nanoseconds utilizing an NVT ensemble to achieve stability of the protein–ligand complexes. A 12 ns brief equilibration and minimization run using an NPT ensemble was executed. The NPT ensemble was established using the Nosé–Hoover chain coupling scheme [119] with a constant pressure of 1 bar throughout all simulations, a variable temperature, and a relaxation time of 1.0 ps. For pressure regulation, the Martyna–Tuckerman–Klein chain coupling scheme [120] barostat method was implemented with a relaxation time of 2 ps and a time step of 2 fs. The Ewald method for long-range electrostatic interactions was implemented using the particle mesh [121], maintaining the coulomb interaction radius at 9 Å. The RESPA integrator was employed to compute bonded forces, utilizing a time step of 2 fs for each trajectory. The ultimate production run was 200 nanoseconds when conducted. To monitor the stability of the MD simulation, parameters including RMSD, rRg, and RMSF were calculated.

#### 4.2.12. Binding Free Energy Analysis of L3 and L4 Complex

The molecular mechanics combined with the generalized born surface area (MM–GBSA) approach was employed to calculate the binding free energies of the ligand–protein complexes. Specifically, the Prime MM–GBSA binding free energy was determined using a Python script named “thermal mmgbsa.py”. The calculations were conducted on the last 50 frames of the simulation trajectory with a 1-step sampling size. The binding free energy of Prime MM–GBSA was expressed in kcal/mol, utilizing the principle of additivity. This methodology considered various individual energy components, encompassing Coulombic interactions, covalent bonds, hydrogen bonding, van der Waals forces, self-contact energy, lipophilic interactions, and solvation of the protein. These diverse energy contributions were amalgamated to yield the overall binding free energy, furnishing valuable insights into the stability and strength of the ligand–protein interactions [122].

## 5. Conclusions

To the best of our knowledge, this study offers the first comprehensive computational approach to address the pressing issue of developing effective antiviral drugs against the Kyasanur Forest Disease virus (KFDV) envelope protein. An integrated computational approach involving molecular modeling, ligand designing, virtual screening, molecular docking, ADMET studies, QM studies, MD simulation, and MM–GBSA techniques revealed two promising compounds, L4 (SA28) and L3 (CNP0247967), with strong binding affinity to the envelope protein and with free energy values of −66.60 ± 2.92 kcal/mol and −85.26 ± 26 kcal/mol, respectively. These compounds outperformed BOG inhibitors as determined by COACH-D in terms of binding energy. This research represents a significant step in the development of effective antiviral drugs for KFDV targeting the envelope protein by a computational approach. Further validation through in vitro assays would complement the findings of the present in silico investigations.

## Figures and Tables

**Table 1:** Homologous sequence of envelope protein by BLASTp.

Sr. No.	Organism	PDB ID	Max Score	Query Cover	Identity %	e-Value
01	Tick-borne encephalitis virus (strain HYPR)	5O6A	851	100%	80.24	0
02	Louping ill virus	6J5C	689	80%	80.8	0
03	Dengue virus 2	4CBF	394	99%	40.99	9.00 × 10^−133^
04	Zika virus	5GZR	377	99%	39.06	1.00 × 10^−125^
05	Japanese encephalitis virus	5WSN	375	99%	39.45	5.00 × 10^−125^

**Table 2:** Physicochemical properties of the envelope protein.

Features	Remark
Protein	Envelope protein
Accession number	D7RF80.1|POLG_KFDV:282–777
Length of sequence	496 aa
pI (theoretical)	7.26
Molecular mass	53,633.08 Da
Index (aliphatic)	83.10
GRAVY	−0.16
Index (instability)(II)	29.41Stable

**Table 3:** The 2D structure of envelope proteins by SOPMA.

Type of Secondary Structure	No. of Amino Acids
Alpha helix (Hh)	95 (19.15%)
Extended strand (Ee)	167 (33.67%)
Pi helix (Ii)	0 (0.00%)
Bend region (Ss)	0 (0.00%)
Random coil (Cc)	203 (40.93%)
Beta turn (Tt)	31 (6.25%)
Beta bridge (Bb)	0 (0.00%)
310 helix (Gg)	0 (0.00%)

**Table 4:** Transmembrane region of envelope protein.

	Location of Domain	Sequence Position
Envelope protein	Extracellular region	1–446
	Transmembrane	447–469
	Cytoplasmic region	470–475
	TM helix	476–495

**Table 5:** Quality assessment of the generated model.

Validation Method	Robetta Model	I-Tasser Model	Refined Model
ERRAT score	89.85	91.51	94.19
Procheck			
Most favored region	93.1%	75.4%	92.3
Additionally allowed regions	6.5%	20.3%	7.2
Generously allowed region	0.2%	3.1%	0.2
Disallowed region	0.2%	1.2%	0.2
ProSA Web score	−7.91	−7.47	−7.56
Verify 3D	89.11%	78.63%	76.81%
QMean Disco Global	0.72 ± 0.05	0.65 ± 0.05	0.73 ± 0.05
QmeanZscore	1.03	−7.11	0.09
Qmean all-atom	0.56	−0.43	0.60
Qmean torsion	1.30	−6.10	0.16
Qmean Solvation	−0.72	−2.78	0.68
Qmean Cβ	−0.09	−1.29	0.80

**Table 6:** The active site of envelope protein predicted by COACH-D.

Predicted Binding Site	c-Value	Docking Energy (kcal/mol)	Active Site Grid Box (x, y, z)	Amino Acid Residue
1	0.09	−4.6	15.707, 0.627, 11.566	Ile^48^, His^49^, Gln^50^, Pro^194^, Val^215^, Val^273^, Ala^274^, Gly^286^
2	0.07	−1.3	11.537, −25.032, −2.164	Asp^149^, Tyr^150^, Asn^154^, Ser^158^, Asn^159^
3	0.07	−5.6	11.120, −9.669, −8.399	Arg^9^, Thr^32^
4	0.05	−4.1	10.638, −25.498, 2.086	Ala^152^, Ser^156^
5	0.04	−3.9	23.933, 10.562, −16.247	Leu^430^, Val^433^, Leu^437^

**Table 7:** Pharmacophore features of BOG.

Pharmacophore Features	Coordinates of Center			Radius (Å)
	x	y	z	
HBD	14.99	3.66	2.38	0.5
HBD	17.58	3.28	3.18	0.5
HBD	18.62	6.15	4.05	0.5
HBD	13.11	4.53	4.83	0.5
HBA	14.99	3.66	2.38	0.5
HBA	17.58	3.28	3.18	0.5
HBA	18.62	6.15	4.05	0.5
HBA	13.11	4.53	4.83	0.5

HBD: hydrogen bond donor; HBA: hydrogen bond acceptor.

**Table 8:** List of drugs selected based on minimum binding energy by PyRx software.

Ligand ID	Formula	Structure	Binding Energy (kcal/mol)
161783612	C_17_ H_30_ O_9_	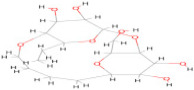	−10.20
CNP0187513.6	C_26_ H_30_ N_2_ O_8_	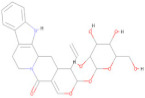	−8.80
CNP0247967	C_27_ H_40_ O_8_	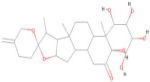	−8.80
SA8	C_25_ H_28_ N_6_ O_2_	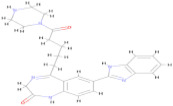	−8.80
101929509	C_14_ H_26_ O_9_	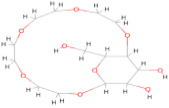	−8.60
ZINC000028541549	C_26_ H_30_ N_2_ O_8_	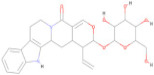	−8.60
ZINC000100052673	C_26_ H_22_ O_10_	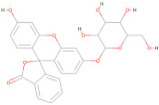	−8.60
CNP0097629.2	C_29_ H_34_ O_9_	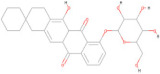	−8.50
CNP0178494.1	C_24_ H_20_ O_12_	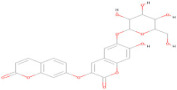	−8.50
CNP0247704.2	C_25_ H_30_ O_9_	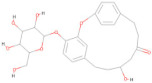	−8.50
SA28	C_33_ H_34_ N_4_ O_3_	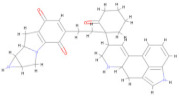	−8.50
SA29	C_33_ H_34_ N_4_ O_3_	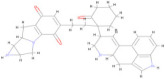	−8.50

**Table 9:** Toxicity measurement of selected drug molecules by ProTox II.

Name of Drug	Oral Toxicity		Organ Toxicity-Hepatotoxicity	Carcinogenicity	Mutagenicity	Cytotoxicity
	LD50	Class				
161783612 (L1)	2000	4	IA	IA	IA	IA
CNP0187513.6 (L2)	300	3	IA	IA	IA	IA
CNP0247967(L3)	10000	6	IA	IA	IA	A
SA8(L4)	2000	4	IA	IA	IA	IA
101929509(L5)	51	3	IA	IA	IA	IA
ZINC000028541549 (L6)	300	3	IA	IA	IA	IA
ZINC0001000052673(L7)	2190	5	IA	IA	IA	IA
CNP0097629.2(L8)	3000	5	IA	IA	IA	IA
CNP0178494.1(L9)	5000	5	IA	IA	A	IA
CNP0247704.2(L10)	4000	5	IA	IA	IA	IA
SA28(L11)	400	4	IA	IA	A	IA
SA29(L12)	400	4	IA	IA	A	IA

IA: Inactive; A: Active.

**Table 10:** ADME analysis of selected drug molecules by SwissADME.

Molecule	161783612 (L1)	SA8 (L4)	CNP0247967 (L3)	ZINC0001000052673 (L7)	CNP0097629.2 (L8)	CNP0247704.2 (L10)
Formula	C_17_H_30_O_9_	C_25_H_32_N_6_O_2_	C_27_H_40_O_8_	C_26_H_22_O_10_	C_29_H_34_O_9_	C_25_H_30_O_9_
Physicochemical properties						
Weight (molecular) (Da)	378.41	448.56	492.6	494.45	526.57	474.5
#Heavy atoms	26	33	35	36	38	34
#Rotatable bonds	2	7	0	3	3	3
#Aromatic heavy atoms	0	12	0	18	6	12
#H-bond donors	5	5	5	5	5	5
#H-bond acceptors	9	4	8	10	9	9
MR	87.64	150.18	126.48	120.87	135.38	120.32
TPSA	138.07	97.53	136.68	155.14	153.75	145.91
Lipophilicity Consensus log P	−0.96	1.54	1.11	1.29	1.83	0.87
Water solubility ESOL log S	−0.96	−3.39	−3.24	−4.1	−4.46	−3.48
Water solubility ESOL class	Very soluble	Soluble	Soluble	Moderately soluble	Moderately soluble	Soluble
Pharmacokinetics						
GI absorption	Low	High	High	Low	Low	Low
log Kp (cm/s) skin permeation	−9.84	−7.96	−8.91	−8.17	−7.89	−8.48
BBB permeant	No	No	No	No	No	No
Drug likeness						
Lipinski #violations	0	0	0	0	1	0
Veber #violations	0	0	0	1	1	1
Bioavailability score	0.55	0.55	0.55	0.55	0.55	0.55
PAINS #alerts	0	0	0	0	1	0
Lead likeness #violations	1	1	1	1	1	1
Synthetic accessibility	7.32	4.06	6.93	5.78	6.72	6.76
Metabolism						
Pgp substrate	Yes	Yes	Yes	No	Yes	Yes
CYP2D6 inhibitor	No	Yes	No	No	No	No
CYP2C9 inhibitor	No	No	No	No	No	No
CYP2C19 inhibitor	No	Yes	No	No	Yes	No
CYP1A2 inhibitor	No	No	No	No	No	No
CYP3A4 inhibitor	No	No	No	Yes	No	No

**Table 11:** Molecular docking results of six compounds by AutoDock.

Compound ID	Name	Binding Affinity (kcal/mol)	Inhibition Constant K_i_
161783612 (L1)	(1S,3R,5R,6S,12R,14R,15R,16R,17R,18R)-5-ethyl-14-(hydroxymethyl)-2,4,7,13-etraoxatricyclo [10.2.2.23,6]octadecane-15,16,17,18-tetrol	−4.58	436.03 µM
CNP0247967 (L3)	Tupichigenin C	−7.26	4.79 µM
SA28 (L4)	De novo design	−7.82	1.86 µM
ZINC0001000052673 (L7)	Fluorescein beta-D-galactopyranoside	−7.65	2.48 µM
CNP0097629.2 (L8)	5′-hydroxy-7′-{[3,4,5-trihydroxy-6-(hydroxymethyl)oxan-2-yl]oxy}-3′,4′,5′a,6′,11′,11′a-hexahydro-1′H-spiro[cyclohexane-1,2′-tetracene]-6′,11′-dione	−7.04	6.91 µM
CNP0247704.2 (L10)	10-hydroxy-4-{[3,4,5-trihydroxy-6-(hydroxymethyl)oxan-2-yl]oxy}-2-oxatricyclo [13.2.2.1^3^,^7^]icosa-1(17),3(20),4,6,15,18-hexane-12-one	−5.72	64.51 µM

**Table 12:** Calculated FMO energy band values of ligands.

Ligand	Optimized Energy (eV)	HOMO (eV)	LUMO (eV)	Energy Gap (eV)
L3	−44,966	−6.63	−0.99	5.63
L4	−39,428	−4.85	−0.66	4.18
L7	−47,727	−6.15	−0.05	4.71
L8	−48,985	−5.15	−1.93	3.20
L10	−44,777	−6.32	−0.95	5.37

**Table 13:** Ligand binding energy values before and after geometry optimization.

Compound ID	Binding Affinity (kcal/mol)	
	Before Optimization	After Optimization
CNP0247967 (L3)	−7.26	−7.58
SA28 (L4)	−7.82	−8.91
ZINC0001000052673 (L7)	−7.65	−6.43
CNP0097629.2 (L8)	−7.04	−6.12
CNP0247704.2 (L10)	−5.72	−6.02

**Table 14:** Interaction analysis of selected ligands with envelope protein.

Complex	Amino Acid Residue	Bond Distance (Å)	Bond Category	Type of Bond
L3	His216	1.95	HB	CHB
	Gly270	1.65	HB	CHB
	Gly270	2.11	HB	CHB
	Val192	2.67	HB	Carbon H–B
	Val215	2.99	HB	Carbon H–B
	His216	2.42	HB	Carbon H–B
	Gln214	2.86	HB	Carbon H–B
	Val192	4.89	HP	Alkyl
	Val192	3.73	HP	Alkyl
	His287	4.19	HP	Pi–Alkyl
	His419	4.34	HP	Pi–Alkyl
L4	Gly191	1.77	HB	CHB
	Gln214	1.75	HB	CHB
	Val415	2.55	HB	CHB
	His216	2.75	HB	Carbon H–B
	His216	2.75	HB	Carbon H–B
	Val192	4.93	HP	Pi–Alkyl
	Val415	5.24	HP	Pi–Alkyl
L7	Gln214	2.10	HB	CHB
	Glu26	2.10	HB	CHB
	Leu27	2.38	HB	CHB
	Gly270	1.67	HB	CHB
	Ser285	2.83	HB	Pi–Donor H–B
	Val192	5.23	HP	Pi–Alkyl
	Pro272	3.90	HP	Pi–Alkyl
L8	Val273	2.39	HB	CHB
	Ser285	2.15	HB	CHB
	Gln284	2.27	HB	CHB
	Val271	2.28	HB	CHB
	Ser285	2.87	HB	Carbon H–B
	Val415	2.77	HB	Carbon H–B
	His287	3.71	HP	Pi–Sigma
	Val192	5.29	HP	Alkyl
	Val192	4.37	HP	Alkyl
	His287	4.93	HP	Pi–Alkyl
	Val415	5.18	HP	Pi–Alkyl
L10	Gln196	2.79	HB	CHB
	Gly191	2.13	HB	CHB
	Asp193	2.68	HB	Pi–Donor H–B
	His216	4.57	HP	Pi–Pi T-shaped
	Val192	4.60	HP	Pi–Alkyl

HB: hydrogen bond; HP: hydrophobic; CHB: conventional H–B.

**Table 15:** Binding free energy components for the L3 and L4 complexes calculated by MM-GBSA.

Energies (kcal/mol)	L3 Complex	L4 Complex
ΔG_bind_	−85.26 ± 4.63	−66.60 ± 2.92
ΔG_bind_Lipo	−32.51 ± 1.64	−19.96 ± 0.96
ΔG_bind_vdW	−70.63 ± 3.57	−53.79 ± 1.23
ΔG_bind_Coulomb	−43.66 ± 4.48	−23.60 ± 1.52
ΔG_bind_H_bond_	−1.87 ± 0.25	−0.58 ± 0.01
ΔG_bind_SolvGB	60.54 ± 3.45	33.55 ± 2.75
ΔG_bind_Covalent	4.22 ± 1.66	0.31 ± 0.64

## Data Availability

The original contributions presented in the study are included in the article/supplementary material, further inquiries can be directed to the corresponding author/s.

## Supplementary Materials

The following supporting information can be downloaded at: https://www.mdpi.com/article/10.3390/ph17070884/s1, Table S1: List of 50 compounds designed by the eLea3d serve (de novo design); Table S2: List of compounds selected for virtual screening from COCONUT and Zinc database; Table S3: Virtual screening of the drugs with minimum binding energy by PyRx software.
