# Supplementary material for: Computational Exploration of Potential Pharmacological Inhibitors Targeting the Envelope Protein of the Kyasanur Forest Disease Virus
Source: Pharmaceuticals (Basel). 2024 Jul 3;17(7):884. doi: 10.3390/ph17070884 (PMC11279457; doi:10.3390/ph17070884)
Supplement: Supplementary file 1 [file pharmaceuticals-17-00884-s001.zip › pharmaceuticals-3051832-supplementary.pdf]

pharmaceuticals

MDPI

Article

Computational Exploration of Potential Pharmacological Inhibitors Targeting the Envelope Protein of the Kyasanur Forest Disease Virus

Table S1: List of 50 compounds designed by the eLea3d serve (*de-novo* design).

Compound ID	Drugs Smile format	Molecular Formula	Molecular Weight (Da)
SA1	C=CC(=O)CCC(=O)N1CCNCC1	C10 H16 N2 O2	196.252
SA2	O=C(CCc1ccccc1)N2CCNCC2	C13 H18 N2 O	218.301
SA3	NCCC[C@H](N)CC(=O)NCC(=O)CCc1ccccc1	C16 H25 N3 O2	291.389
SA4	NCCC[C@H](N)CC(=O)NCC(=O)CCc1ccccc1	C16 H25 N3 O2	291.389
SA5	NCCC[C@H](N)CC(=O)NCCCC(=O)c1ccccc1	C16 H25 N3 O2	291.389
SA6	CNCCON(CCCC(=O)c1ccccc1)C(=O)C[C@@H](N)CCCN	C19 H32 N4 O3	364.482
SA7	CNCCON(CCCC(=O)c1ccccc1)C(=O)C[C@@H](N)CCCN	C19 H32 N4 O3	364.482
SA8	O=C(CCCCC1=NCC(=O)Nc2ccc(cc12)c3nc4ccccc4[nH]3)N5CCNCC5	C25 H28 N6 O2	444.529
SA9	C[C@@H](O)[C@H](C)c1ccc(CCC2=NCC(=O)Nc3ccc(O)cc23)cc1	C21 H24 N2 O3	352.427
SA10	C[C@@H](CC[C@@H]1COc2nc(cn2C1)N3CCN(CC3)C4=NCC(=O)Nc5ccccc45)C[C@H](C)[C@H](O)CC=O	C29 H40 N6 O4	536.666
SA11	C[C@@H](O)[C@H](C)c1ccc(CCC2=NCC(=O)Nc3ccc(O)cc23)cc1	C21 H24 N2 O3	352.427
SA12	C[C@@H](O)[C@H](C)c1ccc(CCC2=NCC(=O)Nc3ccc(O)cc23)cc1	C21 H24 N2 O3	352.427
SA13	C[C@@H](O)[C@H](C)c1ccc(CCC2=NCC(=O)Nc3ccc(O)cc23)cc1	C21 H24 N2 O3	352.427
SA14	C[C@@H](O)[C@H](C)c1ccc(CCC2=NCC(=O)Nc3ccc(O)cc23)cc1	C21 H24 N2 O3	352.427
SA15	C[C@@H](O)[C@H](C)c1ccc(CCC2=NCC(=O)Nc3ccc(O)cc23)cc1	C21 H24 N2 O3	352.427
SA16	C[C@@H](O)[C@H](C)c1ccc(CCC2=NCC(=O)Nc3ccc(O)cc23)cc1	C21 H24 N2 O3	352.427
SA17	C[C@@H](O)[C@H](C)c1ccc(CCC2=NCC(=O)Nc3ccc(O)cc23)cc1	C21 H24 N2 O3	352.427
SA18	C[C@@H](O)[C@H](C)c1ccc(CCC2=NCC(=O)Nc3ccc(O)cc23)cc1	C21 H24 N2 O3	352.427
SA19	C[C@@H](O)[C@H](C)c1ccc(CCC2=NCC(=O)Nc3ccc(O)cc23)cc1	C21 H24 N2 O3	352.427
SA20	C[C@@H](O)[C@H](C)c1ccc(CCC2=NCC(=O)Nc3ccc(O)cc23)cc1	C21 H24 N2 O3	352.427
SA21	C[C@@H](O)[C@H](C)c1ccc(CCC2=NCC(=O)Nc3ccc(O)cc23)cc1	C21 H24 N2 O3	352.427
SA22	C[C@@H](O)[C@H](C)c1ccc(CCC2=NCC(=O)Nc3ccc(O)cc23)cc1	C21 H24 N2 O3	352.427
SA23	CC(C)NC[C@@H](O)COc1ccc2NC(=O)CN=C(c2c1)[C@@]3(CCCCC3=O)c4ccccc4	C27 H33 N3 O4	463.569
SA24	C[C@@H](O)[C@H](C)c1ccc(CCC2=NCC(=O)Nc3ccc(O)cc23)cc1	C21 H24 N2 O3	352.427
SA25	C[C@@H](O)[C@H](C)c1ccc(CCC2=NCC(=O)Nc3ccc(O)cc23)cc1	C21 H24 N2 O3	352.427
SA26	C[C@@H](O)[C@H](C)c1ccc(CCC2=NCC(=O)Nc3ccc(O)cc23)cc1	C21 H24 N2 O3	352.427
SA27	CCCCCCCCC1=NCC(=O)Nc2ccc(cc12)[C@H]3[C@H]4[C@H]5N[C@H]5CN4C6=C3C(=O)C=C(C7CC7)C6=O	C31 H36 N4 O3	512.643
SA28	O=C1CCCC[C@]1(CCC2=CC(=O)C3=C(N4C[C@@H]5N[C@@H]5[C@@H]4C3)C2=O)[C@H]6CN[C@@H]7Cc8c[nH]c9cccc(C7=C6)c89	C33 H34 N4 O3	534.648
SA29	O=C1CCCC[C@]1(CCC2=CC(=O)C3=C(N4C[C@@H]5N[C@@H]5[C@@H]4C3)C2=O)[C@H]6CN[C@@H]7Cc8c[nH]c9cccc(C7=C6)c89	C33 H34 N4 O3	534.648
SA30	CC(C)NC[C@@H](O)[C@@H](OC1=CC(=O)C2=C(N3C[C@@H]4N[C@@H]4[C@@H]3[C@@H]2[C@H]5CCCCC5=O)C1=O)c6ccccc6	C29 H35 N3 O5	505.605
SA31	CCCCCCCCc1ccc2NC(=O)CN=C([C@H]3[C@H]4[C@H]5N[C@H]5CN4C6=C3C(=O)C=C(C7CC7)C6=O)c2c1	C31 H36 N4 O3	512.643

SA32	CCCCCCCCc1ccc2NC(=O)CN=C([C@H]3[C@H]4[C@H]5N[C@H]5CN4C6=C3C(=O)C=C(C7CC7)C6=O)c2c1	C31 H36 N4 O3	512.643
SA33	CC(C)NC[C@@H](O)[C@H](CCc1ccccc1)OC2=CC(=O)C3=C(N4C[C@@H]5N[C@@H]5[C@@H]4[C@@H]3[C@H]6CCCCC6=O)C2=O	C31 H39 N3 O5	533.658
SA34	CC(C)NC[C@@H](O)[C@H](CCc1ccccc1)OC2=CC(=O)C3=C(N4C[C@@H]5N[C@@H]5[C@@H]4[C@@H]3[C@H]6CCCCC6=O)C2=O	C31 H39 N3 O5	533.658
SA35	O=C1CN=Cc2cc(ccc2N1)[C@H]3[C@H]4[C@H]5N[C@H]5CN4C6=C3C(=O)C=C(C7CC7)C6=O	C23 H20 N4 O3	400.43
SA36	CC(C)NC[C@@H](O)COc1cn2c3ccccc3C=N[C@H](c2n1)[C@@]4(CCCCC4=O)c5ccccc5	C29 H34 N4 O3	486.605
SA37	CC(C)NC[C@@H](O)COc1cn2c3ccccc3C=N[C@H](c2n1)[C@@]4(CCCCC4=O)c5ccccc5	C29 H34 N4 O3	486.605
SA38	CC(C)NC[C@@H](O)COc1cn2c3ccccc3C=N[C@H](c2n1)[C@@]4(CCCCC4=O)c5ccccc5	C29 H34 N4 O3	486.605
SA39	CC(C)NC[C@@H](O)COc1cn2c3ccccc3C=N[C@H](c2n1)[C@@]4(CCCCC4=O)c5ccccc5	C29 H34 N4 O3	486.605
SA40	CC(C)NC[C@@H](O)COc1cn2c3ccccc3C=N[C@H](c2n1)[C@@]4(CCCCC4=O)c5ccccc5	C29 H34 N4 O3	486.605
SA41	CC(C)NC[C@@H](O)COc1cn2c3ccccc3C=N[C@H](c2n1)[C@@]4(CCCCC4=O)c5ccccc5	C29 H34 N4 O3	486.605
SA42	CC(C)NC[C@@H](O)COc1cn2c3ccccc3C=N[C@H](c2n1)[C@@]4(CCCCC4=O)c5ccccc5	C29 H34 N4 O3	486.605
SA43	CC(C)NC[C@@H](O)COc1cn2c3ccccc3C=N[C@H](c2n1)[C@@]4(CCCCC4=O)c5ccccc5	C29 H34 N4 O3	486.605
SA44	CC(C)NC[C@@H](O)COc1cn2c3ccccc3C=N[C@H](c2n1)[C@@]4(CCCCC4=O)c5ccccc5	C29 H34 N4 O3	486.605
SA45	CC(C)NC[C@@H](O)COc1cn2c3ccccc3C=N[C@H](c2n1)[C@@]4(CCCCC4=O)c5ccccc5	C29 H34 N4 O3	486.605
SA46	CC(C)NC[C@@H](O)COc1cn2c3ccccc3C=N[C@H](c2n1)[C@@]4(CCCCC4=O)c5ccccc5	C29 H34 N4 O3	486.605
SA47	CC(C)NC[C@@H](O)COc1cn2c(n1)[C@@H](N=Cc3ccccc23)[C@@]4(CCCCC4=O)c5cscn5	C26 H31 N5 O3 S	493.621
SA48	CC(C)NC[C@@H](O)COc1cn2c(n1)[C@@H](N=Cc3ccccc23)[C@@]4(CCCCC4=O)c5cscn5	C26 H31 N5 O3 S	493.621
SA49	CC(C)NC[C@@H](O)[C@H](C)Oc1cn2c3ccccc3C=N[C@H](c2n1)[C@@]4(CCCCC4=O)c5cscn5	C27 H33 N5 O3 S	507.648
SA50	CC(C)NC[C@@H](O)[C@H](C)Oc1cn2c3ccccc3C=N[C@H](c2n1)[C@@]4(CCCCC4=O)c5cscn5	C27 H33 N5 O3 S	507.648
SA51	CC(C)NC[C@@H](O)[C@H](C)Oc1cn2c3ccccc3C=N[C@H](c2n1)[C@@]4(CCCCC4=O)c5cscn5	C27 H33 N5 O3 S	507.648

Table S2: List of compounds selected for virtual screening from COCONUT and Zinc database.

Sr.No.	Compound ID from Coconut Database	Binding Affinity (kcal/mol)	Sr.No.	Compound ID from Zinc Database	Binding Affinity (kcal/mol)
1	CNP0269560.4	-9.00165	1	ZINC000118914382	-8.06736
2	CNP0163873	-8.72669	2	ZINC000004073376	-7.51421
3	CNP0197121.2	-8.62999	3	ZINC000008765855	-7.49763
4	CNP0097629.2	-8.47207	4	ZINC000004722028	-7.4376
5	CNP0101955.1	-8.4675	5	ZINC000257373242	-7.43661

6	CNP0328697	-8.37957	6	ZINC000005811092	-7.36634
7	CNP0087579	-8.33522	7	ZINC000028541549	-7.3413
8	CNP0101076.1	-8.27736	8	ZINC000143132030	-7.27041
9	CNP0126799	-8.27452	9	ZINC001560407710	-7.26917
10	CNP0312293	-8.22535	10	ZINC000008762095	-7.24081
11	CNP0215016	-8.15787	11	ZINC000169682650	-7.23865
12	CNP0272723.2	-8.13286	12	ZINC000100052673	-7.22154
13	CNP0178494.1	-8.11915	13	ZINC001560408799	-7.21746
14	CNP0187513.6	-8.11148	14	ZINC000008381630	-7.18777
15	CNP0365565.7	-8.08699	15	ZINC000004544945	-7.17579
16	CNP0247704.2	-8.08238	16	ZINC000257226440	-7.13171
17	CNP0097869.1	-8.07097	17	ZINC000103011683	-7.12087
18	CNP0101955.2	-8.04122	18	ZINC000005885364	-7.11731
19	CNP0284380.1	-8.02526	19	ZINC000101109305	-7.10215
20	CNP0091591.1	-8.01439	20	ZINC000004028710	-7.05629
21	CNP0424362	-8.00619	21	ZINC000390822384	-7.04736
22	CNP0272687.1	-7.99782	22	ZINC000067911597	-7.04105
23	CNP0271345.2	-7.9919	23	ZINC000005811092	-7.02307
24	CNP0429752.1	-7.99107	24	ZINC000005742784	-7.02066
25	CNP0214456.2	-7.98523	25	ZINC000068267876	-7.00422
26	CNP0320762.1	-7.98163			
27	CNP0206317.1	-7.97183			
28	CNP0135311.1	-7.97177			
29	CNP0126799	-7.97145			
30	CNP0424362	-7.9615			
31	CNP0202154	-7.93831			
32	CNP0142149.1	-7.92542			
33	CNP0310825.1	-7.92137			
34	CNP0228930	-7.8988			
35	CNP0329253.2	-7.89017			
36	CNP0161809.2	-7.88647			
37	CNP0196913	-7.88316			
38	CNP0191900.2	-7.85841			
39	CNP0087579	-7.85779			
40	CNP0421608.2	-7.85132			
41	CNP0097858.1	-7.84816			
42	CNP0318532	-7.83364			
43	CNP0272563	-7.82482			
44	CNP0420988	-7.82275			
45	CNP0252677.3	-7.79911			
46	CNP0147281.1	-7.79611			
47	CNP0090750.2	-7.78409			
48	CNP0252677	-7.75802			
49	CNP0087618	-7.75447			
50	CNP0247967	-7.72347			
51	CNP0351558.1	-7.72308			
52	CNP0408878	-7.71024			
53	CNP0227505.1	-7.70804			
54	CNP0089454.1	-7.69578			

55	CNP0113330	-7.68907			
56	CNP0095088.2	-7.68695			
57	CNP0252677.2	-7.68061			
58	CNP0363834.1	-7.67802			
59	CNP0272563.2	-7.66149			
60	CNP0113330	-7.65855			
61	CNP0261923	-7.65767			
62	CNP0261923	-7.65752			
63	CNP0244603	-7.65702			
64	CNP0422042.2	-7.64467			
65	CNP0142149.2	-7.6419			
66	CNP0126059.2	-7.64188			
67	CNP0328697.1	-7.63625			
68	CNP0395442.2	-7.62946			
69	CNP0376238.1	-7.61653			
70	CNP0345140	-7.61514			
71	CNP0161953.1	-7.61489			
72	CNP0126799	-7.60403			
73	CNP0269560.4	-9.00903			

Table S3. Virtual screening of the drugs with minimum binding energy by PyRx software.

**Sr.No.**	**Ligand**	**Binding Energy (kcal/mol)**
1.	161783612	-10.2
2.	CNP0187513.6	-8.8
3.	CNP0247967	-8.8
4.	SA8	-8.8
5.	101929509	-8.6
6.	ZINC000028541549	-8.6
7.	ZINC000100052673	-8.6
8.	CNP0097629.2	-8.5
9.	CNP0178494.1	-8.5
10.	CNP0247704.2	-8.5
11.	SA28	-8.5
12.	SA29	-8.5
13.	SA35	-8.4
14.	141784345	-8.4
15.	CNP0101955.1	-8.3
16.	CNP0328697	-8.3
17.	CNP0101076.1	-8.3
18.	CNP0215016	-8.3
19.	CNP0126799	-8.2
20.	CNP0196913	-8.2
21.	CNP0142149.2	-8.2
22.	CNP0395442.2	-8.2
23.	CNP0163873	-8.1
24.	CNP0420988	-8.1
25.	ZINC000257373242	-8.1

26.	CNP0091591.1	-8
27.	CNP0429752.1	-8
28.	CNP0142149.1	-8
29.	CNP0228930	-8
30.	CNP0261923	-8
31.	CNP0328697.1	-8
32.	SA36	-8
33.	SA38	-8
34.	SA40	-8
35.	SA42	-8
36.	SA43	-8
37.	SA44	-8
38.	SA45	-8
39.	ZINC000005885364	-8
40.	CNP0284380.1	-7.9
41.	CNP0272687.1	-7.9
42.	CNP0329253.2	-7.9
43.	CNP0227505.1	-7.9
44.	CNP0363834.1	-7.9
45.	CNP0126059.2	-7.9
46.	SA37	-7.9
47.	SA39	-7.9
48.	SA41	-7.9
49.	SA46	-7.9
50.	CNP0197121.2	-7.8
51.	CNP0312293	-7.8
52.	CNP0206317.1	-7.8
53.	CNP0147281.1	-7.8
54.	144397423	-7.8
55.	58445404	-7.8
56.	21342001	-7.8
57.	ZINC000118914382	-7.8
58.	ZINC000004544945	-7.8
59.	CNP0269560.4	-7.7
60.	CNP0272723.2	-7.7
61.	CNP0424362	-7.7
62.	CNP0271345.2	-7.7
63.	CNP0161809.2	-7.7
64.	CNP0087618	-7.7
65.	121430411	-7.7
66.	70141988	-7.7
67.	67043565	-7.7
68.	76618113	-7.7
69.	68054044	-7.7
70.	57811483	-7.7
71.	91859080	-7.7
72.	156615381	-7.7
73.	ZINC000067911597	-7.7
74.	CNP0097858.1	-7.6

75.	CNP0318532	-7.6
76.	CNP0089454.1	-7.6
77.	CNP0376238.1	-7.6
78.	CNP0345140	-7.6
79.	91852058	-7.6
80.	91860988	-7.6
81.	140680080	-7.6
82.	156615652	-7.6
83.	156615651	-7.6
84.	156615295	-7.6
85.	134834083	-7.6
86.	ZINC000005811092	-7.6
87.	ZINC000143132030	-7.6
88.	ZINC000008381630	-7.6
89.	ZINC000005742784	-7.6
90.	CNP0087579	-7.5
91.	CNP0365565.7	-7.5
92.	CNP0101955.2	-7.5
93.	CNP0214456.2	-7.5
94.	CNP0202154	-7.5
95.	CNP0310825.1	-7.5
96.	CNP0421608.2	-7.5
97.	CNP0272563.2	-7.5
98.	69861424	-7.5
99.	91849368	-7.5
100.	ZINC000169682650	-7.5
101.	CNP0408878	-7.4
102.	CNP0113330	-7.4
103.	CNP0422042.2	-7.4
104.	CNP0161953.1	-7.4
105.	SA49	-7.4
106.	130409121	-7.4
107.	ZINC000008765855	-7.4
108.	ZINC000257226440	-7.4
109.	ZINC000068267876	-7.4
110.	CNP0135311.1	-7.3
111.	CNP0272563	-7.3
112.	CNP0351558.1	-7.3
113.	155125952	-7.3
114.	155466185	-7.3
115.	ZINC001560407710	-7.3
116.	ZINC000008762095	-7.3
117.	ZINC000101109305	-7.3
118.	CNP0191900.2	-7.2
119.	CNP0090750.2	-7.2
120.	CNP0095088.2	-7.2
121.	CNP0252677.2	-7.1
122.	143276573	-7.1
123.	70128184	-7.1

124.	70125831	-7.1
125.	ZINC000004073376	-7.1
126.	ZINC001560408799	-7.1
127.	ZINC000390822384	-7.1
128.	CNP0097869.1	-7
129.	CNP0252677	-7
130.	CNP0244603	-7
131.	CNP0320762.1	-6.9
132.	CNP0252677.3	-6.9
133.	ZINC000004722028	-6.8
134.	ZINC000103011683	-6.7
135.	70269163	-6.6
136.	72196695	-6.6
137.	161783613	-6.5
138.	ZINC000004028710	-6.4
139.	70483024	-6.3
